# Single-cell spatial atlas of the aging human breast

**DOI:** 10.1038/s43587-026-01104-3

**Published:** 2026-03-31

**Authors:** Pulkit Gupta, Eric Lee, Neus Masqué Soler, Ellen Schrader, Xiao Qian Wang, Shimrit Mayer, Cristina Flores, Sean Beatty, Andrew Roth, Samuel Aparicio, H. Raza Ali

**Affiliations:** 1 CRUK Cambridge Institute, University of Cambridge, Cambridge, UK; 2 Department of Molecular Oncology, BC Cancer Agency, Vancouver, British Columbia Canada; 3 Department of Pathology and Laboratory Medicine, University of British Columbia, Vancouver, British Columbia Canada; 4 Department of Computer Science, University of British Columbia, Vancouver, British Columbia Canada; 5 Department of Medical Genetics, University of British Columbia, Vancouver, British Columbia Canada; 6 Department of Histopathology, Addenbrookes Hospital, Cambridge, UK

**Keywords:** Systems biology, Computational biology and bioinformatics, Ageing

## Abstract

Breast cancer can develop over a wide age range and tumors in younger women differ from those in older women. Aging alters the spatial context of early tumors and may explain these differences, but breast tissue aging remains poorly characterized. Here, using imaging mass cytometry to profile the spatial expression of 40 proteins, we explore age-related remodeling of normal breast tissues in over 3 million cells from 527 reduction mammoplasties. Aged breast tissue was less cellular and less proliferative for all cell types (epithelial, stromal and immune). Tissue architecture was restructured with fewer heterotypic epithelial cell–cell interactions, far fewer lobules and increased fat. Older tissues had a more inflammatory microenvironment with increased M2 macrophages and granzyme B^+^ T cells, contrasted by younger tissues in which B cells were most enriched. Our multiscale atlas extensively details an unexpected general decline of breast tissue with age and reveals its changing spatial context.

## Abstract (web summary)

The dynamics of breast tissue aging are characterized by imaging mass cytometry of over 500 reduction mammoplasties, revealing nonlinear loss of cellularity and a compositional shift favoring inflammation.

## Main

Over 40 years ago, epidemiologists used the concept of ‘breast tissue age’ to explain breast cancer’s violation of the log–linear age-incidence relationship typical of other cancers^1^. The breast is a hormone sensitive organ subject to dramatic remodeling in step with levels of circulating estrogens. ‘Breast tissue age’ reflected events driving estrogen levels associated with breast cancer risk^1^. A recent high-resolution sequencing study showed, however, that breast epithelium steadily accumulates somatic mutations as it ages^2^. Although hormonal factors such as pregnancy explained some of the variation in mutational burden, the vast majority was explained by chronological age^2^. Past analyses have also found that epithelial proliferation and architecture alter with age^3,4^. Aging therefore plays a major and ongoing role in remodeling breast tissue.

We previously showed that host endocrine signaling in breast tumors is strongly age-dependent and found a similar relationship in normal breast tissue^5^. More recently, we have also shown that copy number mutations in breast epithelium are strongly lineage restricted to luminal cells^6^. This suggests that age-related differences in tumor phenotype are in part explicable by corresponding changes in the breast tissue of their origin. In addition to intrinsic characteristics^7^, the spatial context of a cancer’s originating cell probably plays a critical role in sculpting the developing tumor. Some tumors may, for example, originate in niches permissive of their unchecked proliferation, whereas others may be constrained by stromal and immune cells nearby. Altered intercellular communication is a recognized hallmark of aging^8,9^, supporting the idea that spatial context at a cancer’s inception will differ with age. The changing architecture of the aging breast must therefore correspond to changes in the spatial context of early carcinogenesis but remains poorly characterized.

Recent efforts using single-cell and spatial transcriptomic technologies have charted the cellular content of normal breast tissue with high precision^10–13^. A limitation of these studies was their modest sample size and limited power to detect age-related change. To definitively map the cellular and spatial dynamics of the aging breast, we used imaging mass cytometry (IMC)^14^ to profile the in situ expression of 40 proteins at subcellular spatial resolution in normal breast tissue from 527 reduction mammoplasties linked to age data. We found that aged breast tissue is characterized by a general decline in both cellularity and proliferation across all cell types and has restructured architecture with fewer cell–cell interactions, reduced lobules and vasculature but increased ducts and fat and harbours a more inflammatory microenvironment.

## Results

### Spatial mapping of the aging human breast

**Fig. 1:**
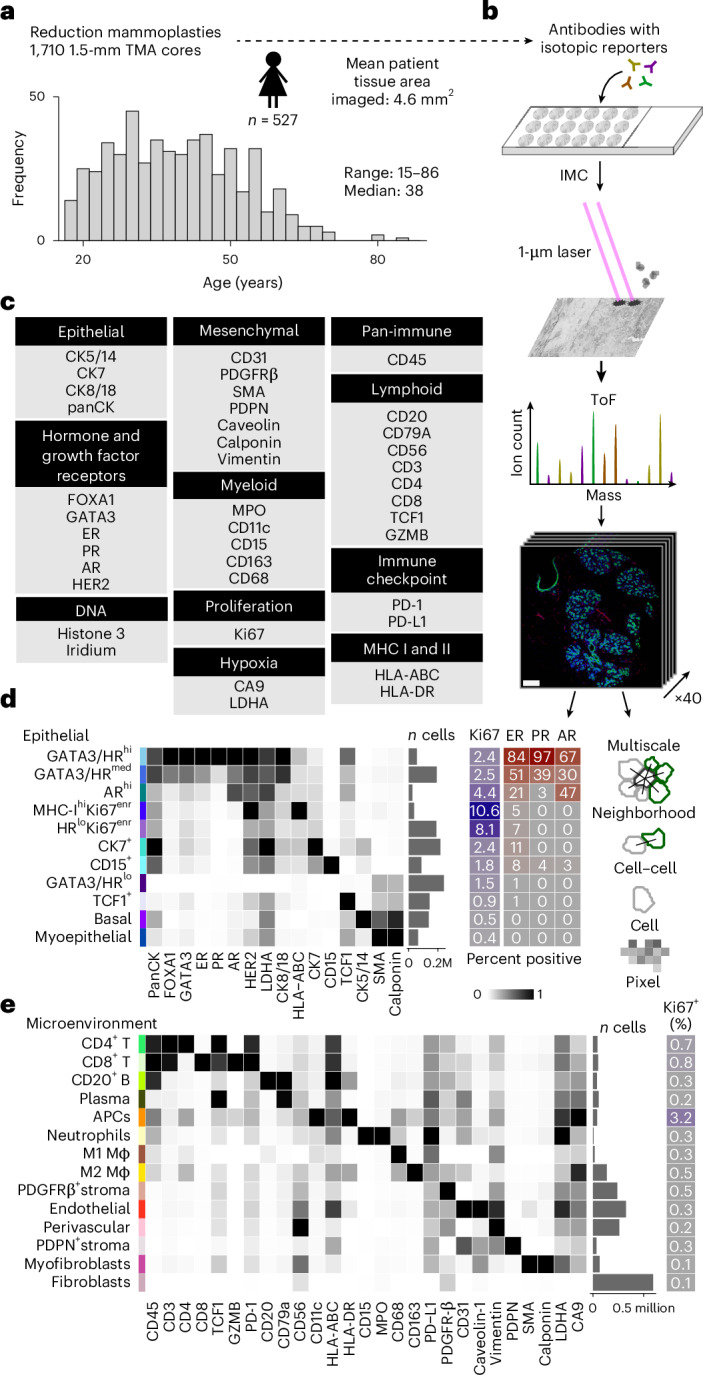
Multiplexed proteomic imaging of the normal breast. **a**, A schematic of the normal breast study with an age distribution histogram. **b**, A flowchart of high-parameter imaging and schematic of analysis levels (scale bar, 150 µm). **c**, Antibody panel targeting 40 markers. **d**, A heat map of median expression values for 11 epithelial cell phenotypes clustered using the proteins on the *x* axis. Right: the bar chart depicts the number of cells per phenotype, and the heatmaps depict positive fractions of markers. **e**, As in **d** for 14 microenvironment cell phenotypes. HR, hormone receptor; Mϕ, macrophage; ToF, time-of-flight.

**Extended Data Fig. 1:**
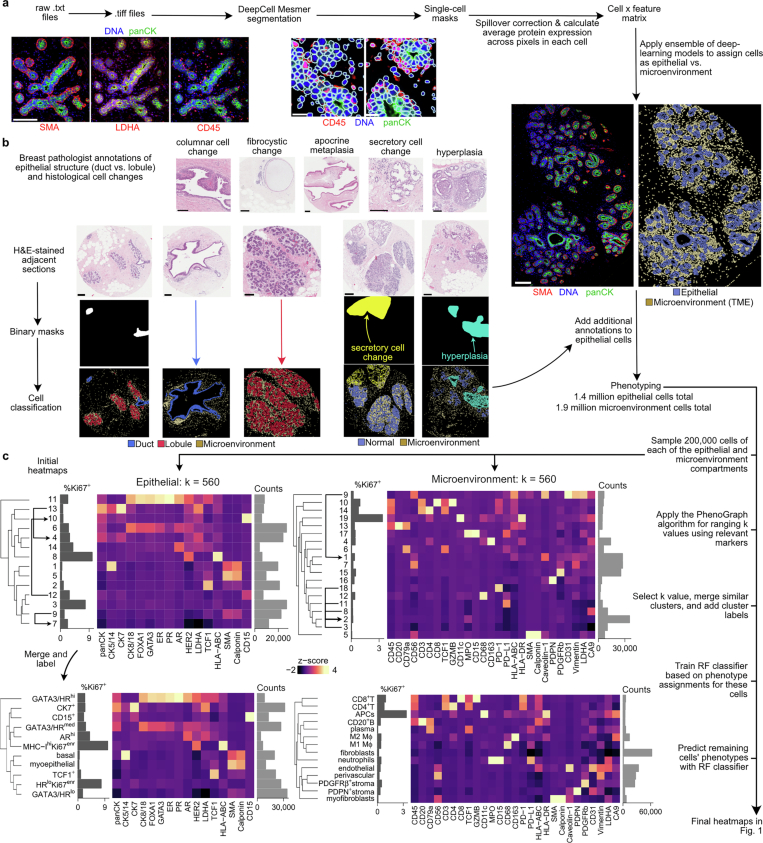
Image processing and phenotyping workflow. Flowchart showing (**a**) image processing and single-cell measurements, (**b**) epithelial histology annotation workflow, and (**c**) detailed phenotyping by compartment. All scale bars, 150 µm, except for the two segmentation image scale bars, 20 µm.

**Extended Data Fig. 2:**
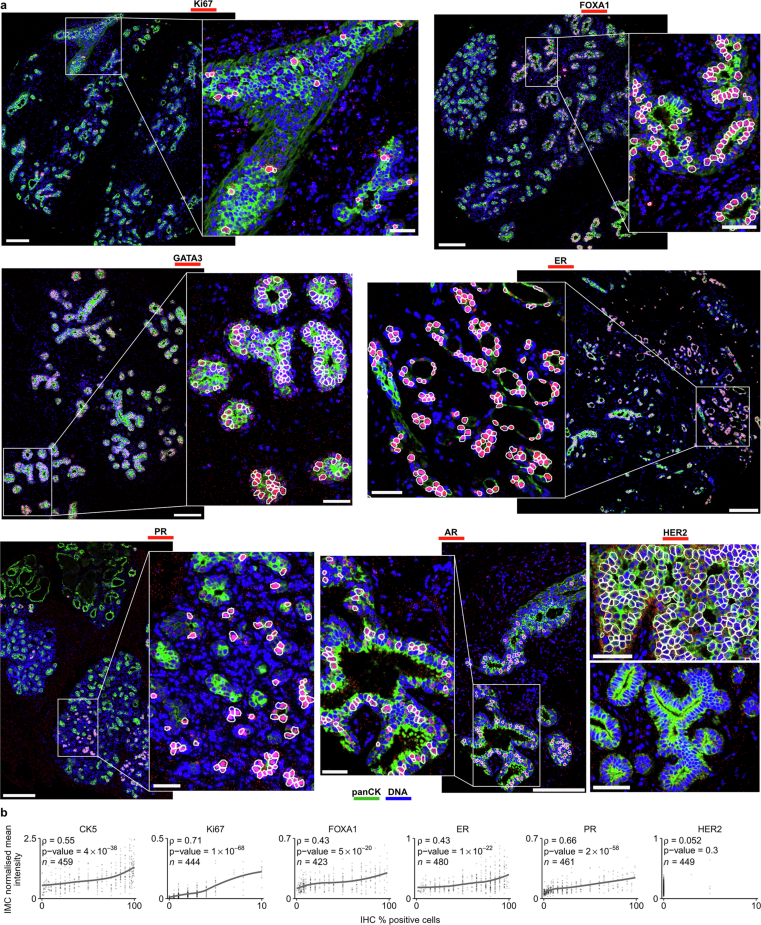
Representative threshold calls for Ki67 and luminal markers and IHC validation of IMC data. **a**, Ki67, FOXA1, GATA3, ER, PR, AR, and HER2-positive cells depicted with white cell borders. Representative images are shown from patients selected from the cohort; similar staining patterns were observed across all patients included in the study. Scale bars for full images, 150 µm; scale bars for zoomed insets and HER2 images, 50 µm. **b**, Relationship between positive cell percentages as assessed by IHC and corresponding IMC normalised mean intensity for 6 markers. Correlation coefficients (ρ) and p-values are derived from two-sided Spearman rank correlation coefficient tests; loess regression lines are shown. IHC, immunohistochemistry; IMC, imaging mass cytometry.

To chart the cellular spatial dynamics of aging breast tissue, we used IMC to profile in situ expression of 40 proteins in a sample collection from a previously reported cohort^5^ of 527 women undergoing reduction mammoplasty and other noncancer related breast surgeries over a wide age range (range 15–86 years, median 38 years; Fig. 1a–c) with tissues sampled as tissue microarrays (TMAs). We assembled a panel of antibodies targeting epitopes critical to breast epithelial lineage commitment and function and key stromal and immune cell types (Fig. 1c). IMC uses laser ablation and time-of-flight mass spectrometry to quantify isotopic reporters conjugated to antibodies with subcellular spatial resolution, generating a stack of images corresponding to each antibody in our panel (Fig. 1b and Supplementary Tables 1 and 2). We processed multiplexed images as single-cell data to derive high-fidelity expression profiles (Extended Data Fig. 1) and used pixel-based clustering to classify cells as positive or negative for key markers such as the proliferation marker Ki67 (Extended Data Fig. 2a and Supplementary Fig. 1). We compared our quantitative single-cell data with past immunohistochemistry (IHC) data for six markers and observed moderate to strong correlations, supporting the validity of our IMC data^5^ (Extended Data Fig. 2b). Histological architecture and benign breast changes were also meticulously annotated under the supervision of a breast pathologist (H.R.A.) (Extended Data Fig. 1). Single cells were classified into 11 epithelial and 14 tissue microenvironment (TME) cell types using data-driven clustering and manual curation (Fig. 1d,e and Supplementary Figs. 2 and 3). Epithelial cells fell within three broad subtypes previously reported in breast atlases: hormone sensing, alveolar and basal^10,15^. Three phenotypes were characterized by hormone receptor expression and subclassified by PR and AR. The most proliferative epithelial cell types were low for hormone receptors and one expressed higher levels of MHC-I (highest proliferation fraction of 10.6%; Fig. 1d). Past work has shown that breast epithelial cells positive for ER do not express proliferation markers^16,17^; we therefore investigated the relationship between proliferation and ER status at single-cell resolution. This analysis showed that that most proliferating epithelial cells (81%) are negative for ER and only a minority (19%) are positive for ER. Since ER^+^ cells were a minority in our data, we also computed the proportion proliferating by ER status (Supplementary Table 3). This revealed that 3.6% of ER^+^ and 2.7% of ER^−^ cells are positive for Ki67. Further exploratory analysis revealed that this preference for proliferation among ER^+^ cells was attributable to cells located in lobules. Of ER^+^ cells in lobules, 5% were proliferating versus 3.3% of ER^−^ cells. In ducts, however, most of this difference was lost: 2.3% of ER^+^ and 2.0% of ER^−^ ductal cells were proliferating. We also found that, on average, among ER^+^ cells, proliferating cells express less ER (Supplementary Fig. 4). These findings indicate that although most proliferating cells in normal breast tissue are ER^−^, the likelihood of proliferating for an individual cell is greatest if it is ER^+^ and located in a lobule and, secondly, that proliferating ER^+^ cells express lower levels of ER. These findings are at odds with past research which showed that expression of ER and Ki67 are mutually exclusive in normal breast tissue^16,17^. The basis of this discordance is unclear but is probably due to several differences in study design: we sourced normal breast tissue from reduction mammoplasties, used a different and possibly more sensitive antibody for ER^18,19^, used an isotopic reporter rather than fluorophore or chromogen and we were able to utilize state-of-the-art segmentation to precisely quantify over 1.3 million epithelial cells together with pixel-based clustering to call positivity rather than manual counting.

**Extended Data Fig. 3:**
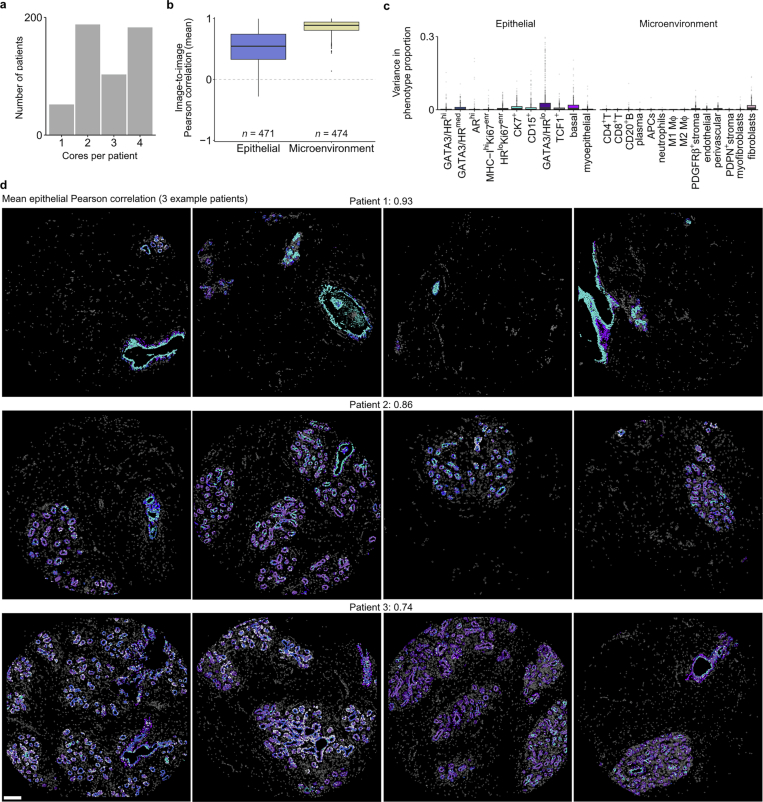
Variation in core-to-core sampling. **a**, Number of cores per patient. **b**, Boxplots of the mean Pearson’s correlation coefficients of cell phenotype proportions for epithelial and microenvironment cell phenotypes, per patient. **c**, Boxplots of the variance in cell phenotype proportions across imaging cores, computed per patient. Each data point represents one patient (biological replicate), with variance calculated across multiple imaging cores from the same patient (technical replicates). For all boxplots, boxes show 25th, 50th, and 75th centiles; whiskers indicate 75th centile ± 1.5 × IQR; points beyond whiskers are outliers. **d**, Mean epithelial phenotype correlation for three example patients with four cores sampled for each patient. All epithelial phenotypes have a unique colour; all other cells are outlined in grey. Scale bar, 150 µm.

Expression of CD15 localized to the luminal surface of ducts and lobules and was occasionally seen within intraluminal secretions; one cell type was characterized by high CD15 (Supplementary Fig. 2). There were two cell types that expressed SMA and calponin (markers of myoepithelial cells) at high levels: one was characterized by high expression of basal cytokeratins (CK5/14), labeled basal, and the other was labeled myoepithelial (Fig. 1d). These two phenotypes were distinguished by their location: the basal phenotype was markedly enriched in ducts whereas the myoepithelial phenotype (lacking expression of CK5/14) was enriched in lobules (Supplementary Fig. 5). Note that these labels (basal and myoepithelial) are based on expression profile, morphology and spatial location and should not, therefore, be used to draw inferences about the replicative capacity or stemness of these cell phenotypes. Likewise, other cell labels were not validated using functional assays; hence, it is possible that these descriptive labels do not entirely capture cell characteristics and approximate cell identity. Among TME cells, major subtypes of lymphoid and myeloid cells were readily identified using established markers of lineage (Fig. 1e). Platelet-derived growth factor receptor-β (PDGFRβ) and podoplanin subclassified two stromal cell types (Fig. 1e and Supplementary Fig. 3). As most tissue samples were represented by more than one TMA spot (Fig. 1a and Extended Data Fig. 3a), we explored core-to-core spatial heterogeneity with respect to the composition of cell phenotypes by computing correlation statistics between spots from the same individual. We found good-to-excellent correlation between images from the same organ, although, on average, this was lower for epithelial (median Pearson correlation of 0.54) versus microenvironment cells (median Pearson correlation of 0.89), consistent with our observation that variance in phenotypic proportions was greater among epithelial phenotypes (Extended Data Fig. 3b,c). In addition, there were relatively few outliers, indicating that our sampling strategy robustly captured regional spatial variation in normal breast tissue (Extended Data Fig. 3b). In summary, our spatial proteomic workflow yielded a highly curated dataset of 1,710 high-plex images containing 3.3 million cells spanning the epithelium and microenvironment of normal breast tissues.

### Hormone receptor expression increases with age

**Fig. 2:**
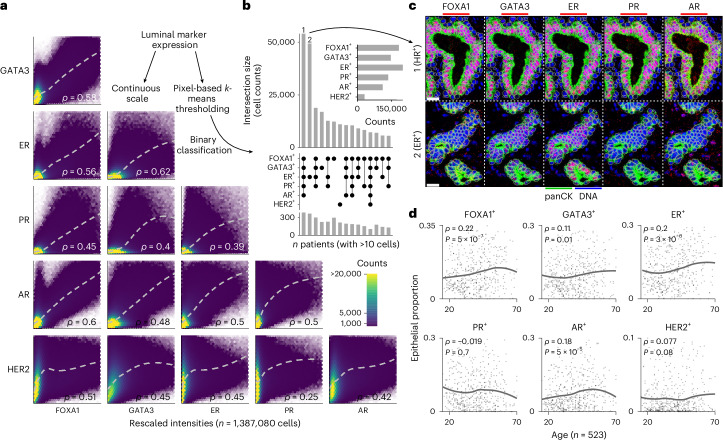
Luminal heterogeneity of the breast epithelium. **a**, Hex density plots of rescaled raw intensities between luminal markers. *ρ* is the Spearman rank correlation coefficient; loess regression lines are depicted. **b**, UpSet plot of luminal marker combination frequencies and numbers of patients with more than ten cells of each combination. Top right: the bar plot is the total cell counts positive for each marker. **c**, Representative images of the top two populations in the bar plot from **b**; scale bar, 20 µm. Representative images are shown from patients selected from the full cohort; similar staining patterns were observed across all patients included in the analysis. **d**, The relationship between proportion of positive luminal marker fraction and age. Correlation coefficients (*ρ*) and *P* values are derived from two-sided Spearman rank correlation coefficient tests, with loess regression lines shown.

**Extended Data Fig. 4:**
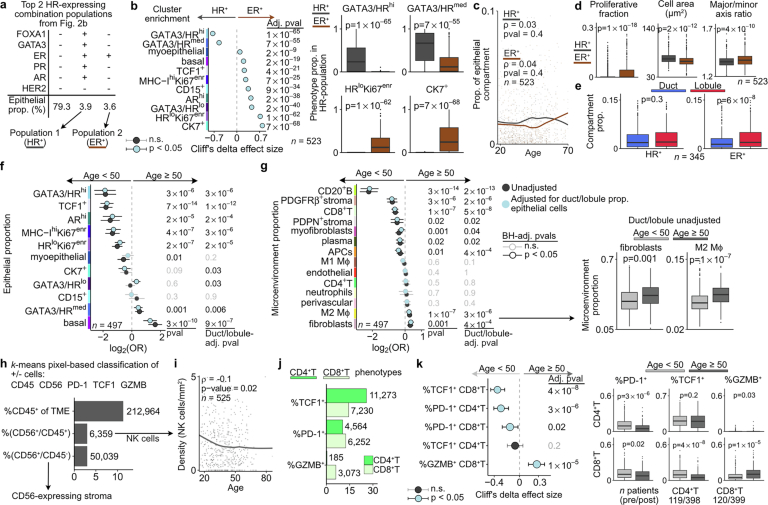
Combined analysis of ER^+^/FOXA1^−^/GATA3^−^ cells, duct/lobule-adjusted phenotypes, and NK/T cell composition. **a**, Identification of HR^+^ (FOXA1^+^/GATA3^+^/ER^+^/PR^+^/AR^+^) and ER^+^ only populations. **b**, Effect sizes for associations between HR-populations and epithelial phenotype presence (p-values from two-sided paired Wilcoxon signed-rank tests) with right-side boxplots showing HR phenotype proportions. **c**, Relationship between HR-population proportion of epithelial compartment and age (two-sided Spearman rank correlation coefficient tests performed). **d**, Boxplots comparing proliferative fraction, cell area, and axis ratio between HR^+^ and ER^+^ cells (paired Wilcoxon tests). **e**, Boxplots of duct vs. lobule assignment of HR^+^ and ER^+^ populations; p-values are from paired Wilcoxon tests from patients with both ductal and lobular cells. **f**, Odds ratios for epithelial phenotype proportions based on an age cutoff of 50, adjusted for duct/lobule epithelial cell proportions in each image (unadjusted and adjusted estimates shown, 95% CIs, p-values from two-sided binomial generalised linear mixed-effects models with Benjamini-Hochberg correction). **g**, As in **f**, for microenvironment phenotypes. **h**, Bar plots of cells expressing CD45 and/or CD56. **i**, Relationship between NK cell density and age, as in **c**. **j**, Bar plots of TCF1, PD-1, and GZMB status for CD4^+^T and CD8^+^T phenotypes. **k**, Effect sizes for associations between marker positivity of the CD4^+^T or CD8^+^T compartment and age (two sided Mann-Whitney U tests, BH-corrected, with right-side boxplots of compartment proportions). For all effect size plots, circles represent point estimates corresponding to median differences between groups, and whiskers indicate 95% confidence intervals with p-values corrected for multiple testing with the BH method. For all scatterplots, correlation coefficients (ρ) and p-values are derived from Spearman rank correlation coefficient tests; loess regression lines are shown. For all boxplots, boxes show 25th, 50th, and 75th centiles; whiskers indicate 25th/75th centiles ± 1.5 × interquartile range; points beyond whiskers are outliers.

Breast tissue is subject to fluctuating levels of estrogens as individuals age and this is known to affect epithelial cell states^1^. Previous studies have identified distinct ER^+^ and PR^+^ cell lineages, including a PR^lo^ subtype enriched in *BRCA2*-mutant carriers^15^. We therefore first explored expression patterns of six hormone receptors and related proteins (ER, PR, AR, FOXA1, GATA3 and HER2) among 1.4 million epithelial cells (Fig. 2). All six markers were positively correlated, with the most strongly correlated pairs being ER and GATA3 (*ρ* = 0.62) and FOXA1 and AR (*ρ* = 0.6) (Fig. 2a). To characterize expression profiles further, we determined whether cells were positive or negative for each marker using pixel-based clustering (Fig. 2b and Extended Data Fig. 2a). This showed that ER and FOXA1 were the most widely expressed markers, and only a small minority of cells were positive for HER2. Importantly, the vast majority of epithelial cells (1.1 million, 79%) were negative for all six markers. Combinatorial expression patterns were highly heterogeneous across all cells (Supplementary Fig. 6), but two cell populations were dominant: cells positive for all five hormone-related proteins (HR^+^) were most abundant (3.9%), and cells only positive for ER (ER^+^) were second most abundant (3.6%) (Fig. 2b,c). Comparing these two populations revealed five-marker HR^+^ cells were concordantly enriched among phenotypes characterized by high HR protein expression, whereas those in the ER^+^ population were most enriched among the CK7^+^ phenotype (Extended Data Fig. 4a,b). Notably neither population varied with age but they did differ in their activation state and morphology: HR^+^ cells were less proliferative, slightly larger and more rounded. Their microanatomic location also differed: ER^+^ cells were enriched in lobules relative to ducts, whereas HR^+^ cells were not (Extended Data Fig. 4c–e). We also investigated the relationship between age and the proportion of epithelial cells that were independently positive for each of the six markers. There was a slight positive correlation between the proportion of cells positive for ER, AR, FOXA1, GATA3 and age, but the proportion of PR^+^ cells did not significantly change with age (Fig. 2d). These analyses reveal marked heterogeneity for hormonal signaling and a steady accumulation of cells positive for hormone related proteins as women age.

### Density of most cell types declines with age

**Fig. 3:**
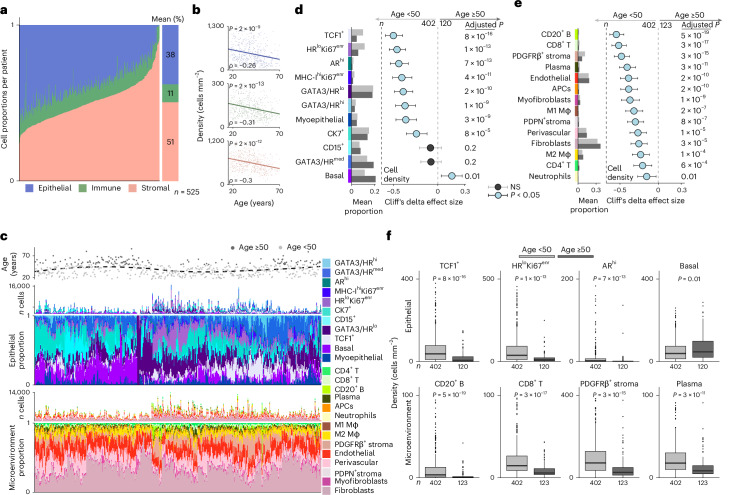
Phenotypic composition of the normal breast. **a**, The proportion of epithelial, immune and stromal cells per patient. **b**, Cellular density as a function of age for each cellular compartment. Correlation coefficients (*ρ*) and *P* values are derived from two-sided Spearman rank correlation coefficient tests; the linear model line is shown. **c**, The number and proportion of cell phenotypes by compartment (epithelial or microenvironment) are depicted for each patient, along with age. Rolling average of age is shown as a loess line. **d**, Effect sizes for associations between epithelial cell density and age (patients aged <50 versus ≥50 years; *n* shown per group). The circles represent Cliff’s delta point estimates and whiskers indicate the 95% confidence intervals. The *P* values are derived from two-sided Mann–Whitney *U* tests performed at the patient level and corrected for multiple testing with the Benjamini–Hochberg method. Left: the bar charts show the mean epithelial phenotype proportions across patients. **e**, Same as **d** for microenvironment cell densities and proportions (patient-level analysis). **f**, The boxplots of cell densities for selected phenotypes with statistical tests from **d** and **e**. The boxes show 25th, 50th and 75th centiles; the whiskers indicate 25th/75th centiles ± 1.5× interquartile range; points beyond whiskers are outliers. Beneath the boxes are the absolute numbers of patients in each category. NS, not significant.

We next investigated the age-related dynamics of all 25 cell phenotypes (Fig. 1d,e). Although proportions varied considerably between individuals, on average, over half of cells were stromal (51%), next most abundant were epithelial (38%) and a minority were immune (11%) (Fig. 3a). Remarkably, the density (number of cells per square millimeter) of epithelial, stromal and immune cells all declined with age at comparable rates (Fig. 3b). Ordering samples according to their cellular composition illustrated a similar relationship between cellularity and age (Fig. 3c). We mapped age-related changes among cell phenotypes by comparing their densities in young versus old tissue. To distinguish younger from older tissues, we used a cut-off of age 50 years as an approximate surrogate for menopause^20^. Among epithelial cells, only the basal phenotype had a higher density in older tissues (Fig. 3d), whereas the densities of all TME phenotypes were higher in younger tissues with CD8^+^ T cells and B cells showing the largest effects (Fig. 3e,f). Although we found a general decline in cellularity, our findings suggested that the rate of decline may differ for each cell phenotype which would change phenotypic composition. We therefore sought evidence of an age-related compositional shift by testing for associations between the proportion of each cell phenotype and age using linear models (Extended Data Fig. 4f,g). This analysis corroborated our findings, again revealing the epithelial basal phenotype to be most enriched in older tissues. Likewise, in the TME, B cells emerged as by far the most enriched cell phenotype in the young. Although the density of M2 macrophages and fibroblasts was lower in older breast tissue (Fig. 3e), they comprised a higher proportion of TME cells in older tissues (Extended Data Fig. 4g).

We next characterized lymphocyte activation states using pixel-based clustering (Extended Data Fig. 4h). We found natural killer cells were very rare and their density slightly decreased with age consistent with our other findings (*ρ* = −0.1) (Extended Data Fig. 4i). Using TCF1, PD-1 and GZMB to subclassify CD4 and CD8 T cells, we found that CD8^+^TCF1^+^ and CD4^+^PD-1^+^ cells were enriched in the young (relative to total CD8^+^ and CD4^+^ populations), but that CD8^+^GZMB^+^ T cells were increased in aged tissues (Extended Data Fig. 4j,k).

Our analysis revealed a near universal decline in cellular density in aging breast tissue with a concurrent compositional change favoring inflammation in the TME.

### Proliferation diminishes with age

**Fig. 4:**
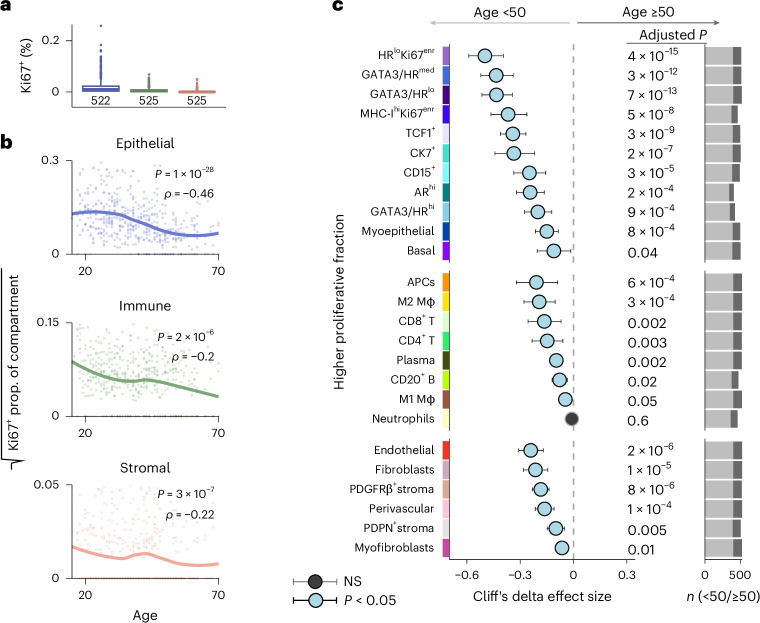
Aging is associated with breast cell-level changes in proliferation and morphology. **a**, Boxplots of proliferative cell fractions for each cellular compartment. The boxes show the 25th, 50th and 75th centiles; the whiskers indicate the 25th/75th centiles ± 1.5× interquartile range; the points beyond whiskers are outliers. Beneath the boxes are absolute numbers of patients in each category. **b**, Ki67^+^ proportion as a function of age for the compartments in **a**. Correlation coefficients (*ρ*) and *P* values are derived from two-sided Spearman rank correlation coefficient tests; loess regression lines are shown. **c**, Effect sizes for associations between proliferative fraction and age (patients aged <50 versus ≥50 years). The circles represent Cliff’s delta point estimates (median difference between groups) and whiskers indicate 95% confidence intervals. The *P* values are derived from two-sided Mann–Whitney *U* tests and corrected using the Benjamini–Hochberg method. Right: the bar plots show the number of patients in each age group (biological replicates) (see Supplementary Table 4 for exact values).

Finding reduced cellularity in aged tissues prompted us to investigate whether this could be explained by less cell proliferation. We used Ki67 to determine the proportion of proliferating cells separately for epithelial, stromal and immune compartments. The overall proportion of proliferating cells was low: epithelial cells were most proliferative (mean of 1.9% Ki67^+^), followed by immune cells (mean of 0.6% Ki67^+^), whereas stromal cells were least proliferative (mean of 0.2% Ki67^+^). Despite the scarcity of proliferating cells, their incidence fell within all three compartments. This reduction was greatest among epithelial cells (*ρ* = −0.46), followed by similar rates in immune (*ρ* = −0.2) and stromal cells (*ρ* = −0.22) (Fig. 4a,b and Supplementary Fig. 7a). We asked whether this reduced proliferation affected different cell phenotypes disproportionately. Remarkably, of the 25 phenotypes we had defined, every one (except neutrophils) showed evidence of an age-related decline in proliferation (Fig. 4c and Supplementary Fig. 7b). Among epithelial cells, phenotypes showing the greatest reduction in proliferative fraction tended to have low levels of HR expression. However, the basal phenotype (low in HR) was associated with the smallest reduction in keeping with their increased prevalence in aged tissue. Despite their marked enrichment in the young, B cells only showed a modest difference in proliferative fraction suggesting other mechanisms play a greater role in regulating their numbers (Fig. 4c). Declining proliferation therefore in part accounts for reduced cellularity in aged breast tissues.

### Stromal cells shrink with age

**Extended Data Fig. 5:**
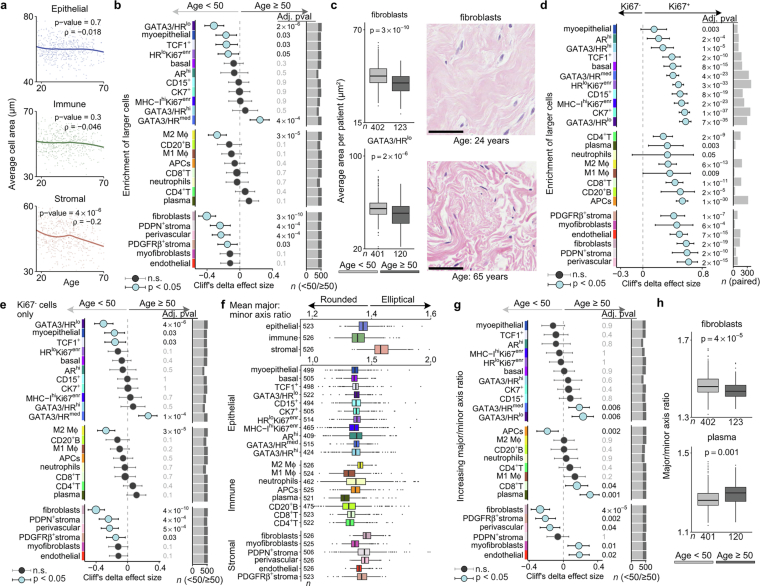
Cell area and axis ratio highlight age-related differences in phenotype morphologies. **a**, Mean cell area as a function of age for each cellular compartment. Correlation coefficients (ρ) and p-values are derived from two-sided Spearman rank correlation coefficient tests; loess regression lines are shown. **b**, Effect sizes for associations between cell size and age: circles represent point estimates corresponding to median differences between groups and whiskers indicate 95% confidence intervals. Depicted p-values are derived from two-sided Mann-Whitney U tests and are corrected for multiple testing with the Benjamini-Hochberg method. Right-sided bar plots show the number of patients in each age group included in analyses (exact values provided in Supplementary Table 4). **c**, Boxplots of mean cell area for selected phenotypes. Representative H&E sections from two different patients are shown; scale bar, 50 µm. **d**, As for **b**, for associations between cell size and Ki67 status. Depicted p-values are derived from two-sided paired Wilcoxon signed-rank tests and are corrected for multiple testing with the Benjamini-Hochberg method. **e**, As for **b**, for Ki67- cells only. **f**, Boxplots of compartment and phenotype mean major/minor axis ratios. **g**, As for **b**, for associations between axis ratio and age. **h**, Boxplots of mean axis ratio for selected phenotypes. For **c**, **f**, **h**, boxes show 25th, 50th, and 75th centiles; whiskers indicate 25th/75th centiles ± 1.5 × interquartile range; points beyond whiskers are outliers. Adjacent to boxes are absolute numbers of patients in each category.

We speculated that changing proliferative fractions reflected altered global activation states that may impact cell morphology. We therefore asked whether cells change shape as breast tissue ages. There was no correlation between cell size and age (as a continuous variable) for epithelial or immune cells but as breast tissue aged stromal cells steadily shrank (*ρ* = −0.2; Extended Data Fig. 5a). By comparing young (<50 years) to older tissues, we found that four epithelial phenotypes were also smaller in older tissue, and one was larger. Among immune cells, only M2 macrophages significantly differed in older versus younger tissues, also showing a reduction in size. Four (of six) stromal phenotypes showed evidence of age-associated shrinking (Extended Data Fig. 5b,c). Given our evidence of reduced cell proliferation with age, we wondered whether age-related differences in cell size could be explained by incidence of dividing cells. Supporting this idea, we found that Ki67^+^ cells were larger than Ki67^−^ cells in all three compartments (epithelial, immune and stromal; Supplementary Fig. 7c). We checked this finding by comparing morphologies across cell types. Epithelial cells tended to be largest, followed by immune and stromal cells (Supplementary Fig. 7c,d). We also confirmed that the proliferating fraction of every cell phenotype was larger than the non-proliferating fraction (Extended Data Fig. 5d). Maintenance of cell size is an important facet of normal tissue homeostasis^21,22^. We reason that that to maintain cell size, a cell would on average need to double in size before dividing, and this probably explains the relationship between division and size that we observe. Having discovered that proliferating cells tended to be larger, we investigated whether the association between reduced cell size and age was preserved among non-proliferating cells and found that it persisted for most phenotypes (Extended Data Fig. 5e). We followed this analysis by asking about cell ellipticity (based on the major:minor axis ratio; Extended Data Fig. 5f). We confirmed the fidelity of our cell morphology data by showing that stromal cells were most elliptical, immune cells least elliptical while epithelial cells fell in-between. Among immune cells, plasma cells were most rounded and M2 macrophages were most elliptical. Notably, among stromal cells, fibroblasts were most elliptical and myofibroblasts were most rounded, linking rounding to cell activation. We explored whether cell ellipticity changes with age. Two epithelial phenotypes (characterized by hormone receptor expression), CD8^+^T cells, plasma cells, myofibroblasts and endothelial cells were all more elliptical in older tissues. Fibroblasts, PDGFRβ^+^ stroma and perivascular cells were, by contrast, more elliptical in younger tissue (Extended Data Fig. 5g,h). Notably, these analyses rely on accurate segmentation of single cells. We used a state-of-the-art segmentation method^23^, and manual inspection of images confirmed excellent performance. Common to all cell segmentation methods, however, is the challenge of defining the bounds of a cell in the absence of a robust and ubiquitous marker of the cell membrane. Segmentations probably underestimate cell size owing to this limitation. We note, however, that past research based on quantitative pathology also uncovered an age-related reduction in nuclear size in human breast tissues^24^, supporting our observations. We conclude that declining cell populations are accompanied by altered cell morphology; most strikingly, stromal cells shrink with age.

### Fewer heterotypic cell–cell interactions with age

**Fig. 5:**
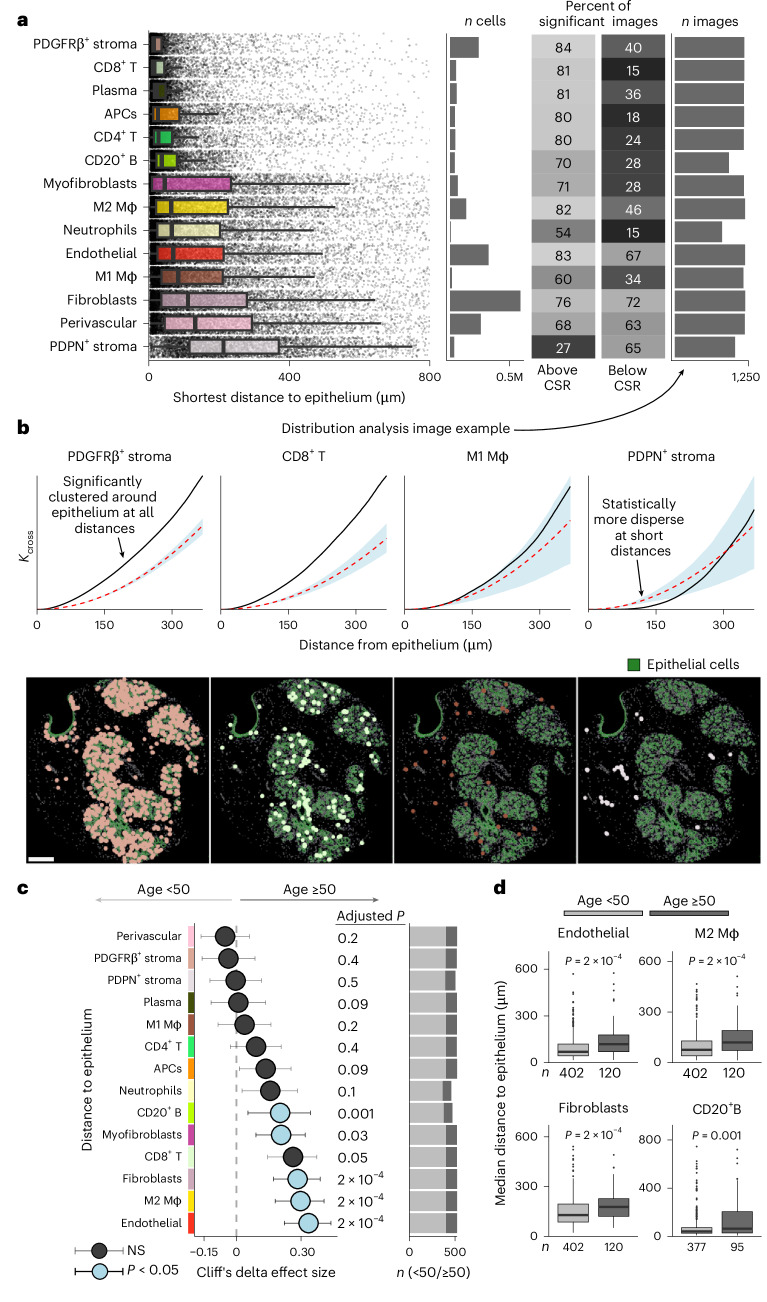
Breast microenvironment spatial structure. **a**, Boxplots depicting the shortest distances from microenvironment cells to any epithelial cell. The boxes show the 25th, 50th and 75th centiles; whiskers indicate the 25th/75th centiles ± 1.5× interquartile range; right: the bar plots show the total numbers of cells included. Random samples of 10,000 cells of each phenotype are also plotted for illustration. The fraction of significant images from bivariate Ripley’s *K* analysis and the number of images used in the analysis for each phenotype are also depicted. **b**, Representative image depicting distribution analysis for selected microenvironment phenotypes. Representative images are shown from one patient; similar spatial patterns were observed across all images included in the analysis. Top: the plots display comparisons between observed *K*_cross_ function values (solid black lines) and *K*_cross_ values under CSR (dashed red lines). Shaded confidence envelopes are derived from 199 Monte Carlo simulations of CSR, representing the upper and lower bounds for a significance level of *P* < 0.01 based on two-tailed tests. Bottom: the images depict epithelium in green and corresponding microenvironment phenotypes as point objects; scale bar, 200 µm. **c**, Effect sizes for associations between shortest distances and age: circles represent point estimates (median difference between age groups) and whiskers indicate 95% confidence intervals. The depicted *P* values are derived from two-sided Mann–Whitney *U* tests and are corrected for multiple testing with the Benjamini–Hochberg method. Right: the bar plots show the number of patients in each age group included in analyses (see Supplementary Table 4 for exact values). **d**, Boxplots of median distances for selected phenotypes. Beneath boxes are absolute numbers of patients in each category.

**Extended Data Fig. 6:**
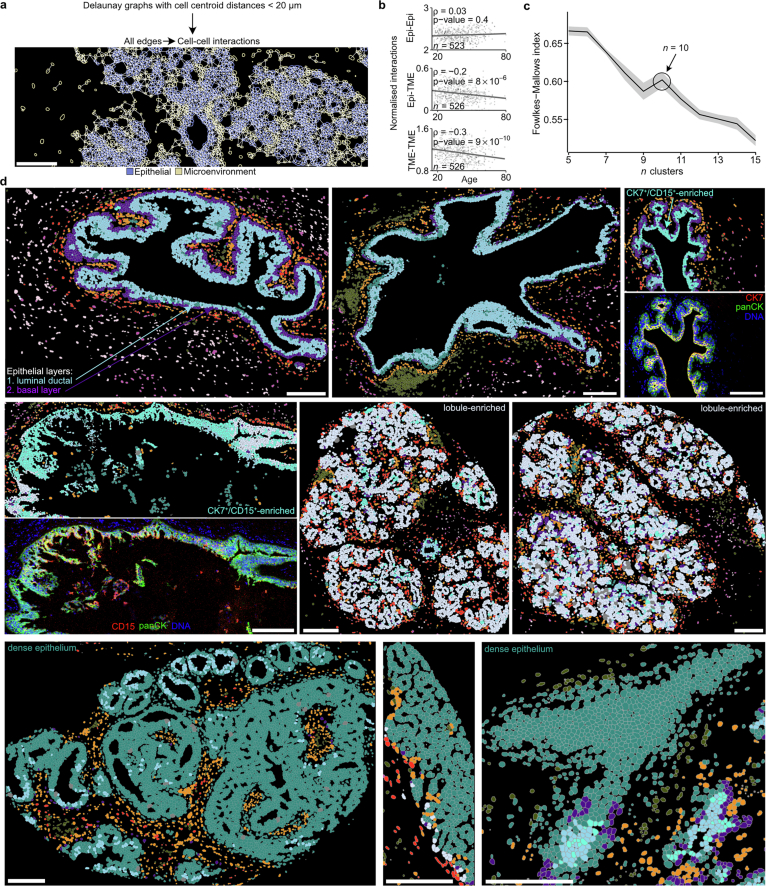
Analysis of adjacent cell-cell interactions and representative epithelial spatial neighbourhood examples. **a**, Schematic of cell-cell interaction identification; scale bar, 100 µm. **b**, Relationship between cell-cell interactions and age; interactions are normalised by dividing interaction counts by the number of cells in the epithelial and/or microenvironment compartments, depending on the interaction type being considered. Correlation coefficients (ρ) and p-values are derived from two-sided Spearman rank correlation coefficient tests; linear regression lines are shown. **c**, Fowlkes-Mallows indices; shaded envelope is the 95% confidence interval from five repetitions for each cluster number. **d**, Depictions of the five epithelial-enriched neighbourhoods; each cell colour represents a unique spatial cluster assignment. Representative images are shown from patients selected from the cohort; similar spatial clustering patterns were observed across all patients analysed. Scale bars, 150 µm.

Age-related change in the spatial context of epithelial cells could impact developing tumors. We therefore characterized the spatial relationships between epithelial and nonepithelial cells and their dynamics with age (Fig. 5). By plotting the number of cell–cell interactions against age, we found that while homotypic epithelial–epithelial interactions did not change over time, epithelial–TME (*ρ* = −0.2) and TME–TME interactions (*ρ* = −0.3) both declined in aged tissue (Extended Data Fig. 6a,b). To explore the spatial context of epithelial cells further, we computed minimum distances between TME and epithelial cells (Fig. 5a,b). This revealed a striking separation of TME cells into two broad groups: those intimately associated with epithelial cells and those more broadly distributed. PDGFRβ^+^ stroma, CD8^+^ T, plasma, antigen-presenting cells (APCs) and CD4^+^ T cells and B cells were all characterized by spatial colocation with epithelial cells. By contrast, all other stromal cells and macrophages showed a much wider distribution. These findings were supported by spatial statistics (bivariate Ripley’s *K*). TME-to-epithelial distances that significantly changed with age, however, involved cells from both groups. Endothelial, M2 macrophages, fibroblasts, myofibroblasts and B cells were all further from epithelial cells in older tissues (Fig. 5c,d). Notably no cell type was significantly closer to epithelium in older tissues. Using spatial statistics, we found aging was associated with fewer epithelial–TME interactions implying that the structural constraints of homeostasis wane with age.

### Age-related change among multicellular neighborhoods

**Fig. 6:**
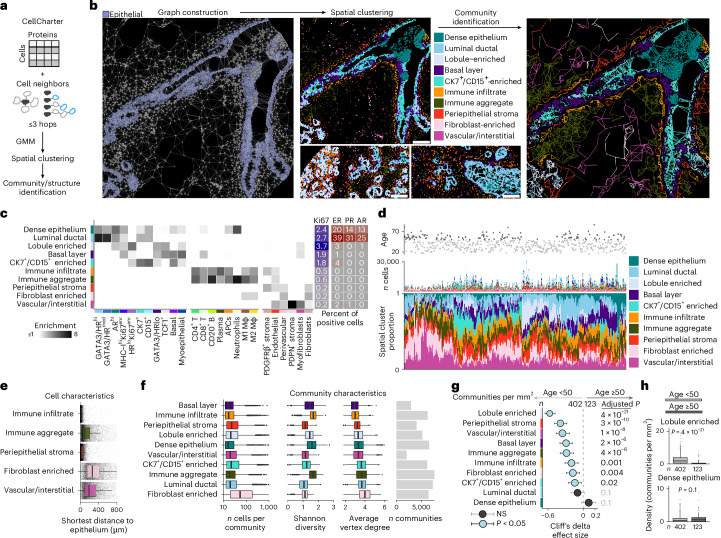
Recurrent multicellular neighborhoods in the normal breast. **a**, A schematic of spatial clustering with CellCharter, followed by network-based community identification. **b**, Representative examples of spatial clustering and structure identification; scale bars, 100 µm. **c**, An enrichment heat map of phenotypes (*x* axis) in neighborhood categories (*y* axis). Enrichments are equivalent to the ratio of observed counts to expected counts; expected counts are based on the proportion of cells in each phenotype and neighborhood. Only enrichments ≥1 are plotted. Right: the heat maps depict positive fractions of markers among total cells. **d**, Number and proportion of cells in each spatial cluster are depicted per patient, along with age. **e**, Boxplots depicting shortest distances from individual spatial-cluster-assigned cells to the nearest epithelial cell. Each data point represents a single cell pooled across all images and patients. Random samples of 10,000 cells per spatial cluster are shown for visualization only and were not used for statistical testing. **f**, Boxplots depicting per-community characteristics, including number of cells, Shannon diversity and mean vertex degree. Each data point represents a single multicellular community (connected component) identified from spatial graphs across all patients. Right: the bar plots indicate the total number of communities contributing to each spatial cluster category. **g**, Effect sizes for associations between community density and age: circles represent point estimates (median difference between age groups) and whiskers indicate 95% confidence intervals. The depicted *P* values are derived from two-sided Mann–Whitney *U* tests and are corrected for multiple testing with the Benjamini–Hochberg method. **h**, Boxplots of community densities for selected spatial clusters. For **e**, **f** and **h**, the boxes show the 25th, 50th and 75th centiles; the whiskers indicate the 25th/75th centiles ± 1.5× interquartile range; the points beyond the whiskers are outliers. Beneath the boxes in **h** are the absolute numbers of patients in each category. Mϕ, macrophage; n.s., not significant.

**Extended Data Fig. 7:**
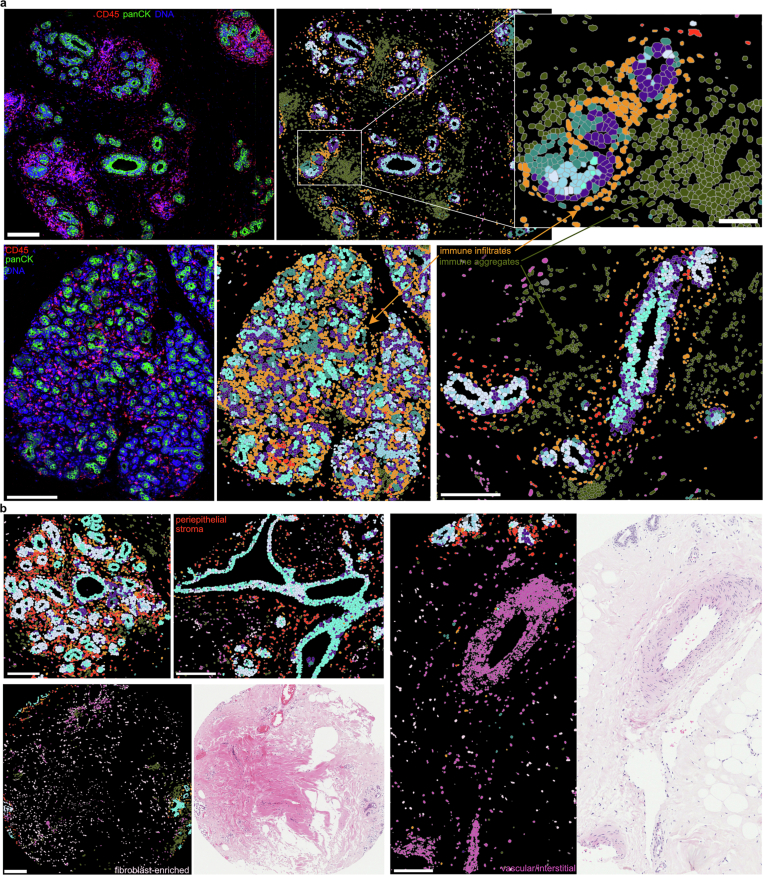
Representative immune and stromal spatial neighbourhood examples. **a**, Depictions of the two immune-enriched neighbourhoods; each cell colour represents a unique spatial cluster assignment. Scale bars, 150 µm; scale bar for zoomed inset, 50 µm. **b**, As for **a**, for the three stromal-enriched neighbourhoods.

We next explored changes in the higher-order organization of breast tissue by aggregating similar cells into discrete groups using CellCharter (Fig. 6a,b and Extended Data Figs. 6 and 7). CellCharter combines the phenotype and location of cells to identify recurrent multicellular neighborhoods based on spatial graphs of cell–cell interactions^25,26^. We defined ten neighborhoods: five were dominated by epithelial and five by TME cells (Fig. 6c,d). Of the TME neighborhoods, two were primarily composed of leukocytes: immune infiltrate (closer to epithelium) and immune aggregate (further from epithelium; Fig. 6e). Immune aggregates were characterized by presence of neutrophils, enrichment for B cells and higher cellular heterogeneity (measured by Shannon diversity; Fig. 6c,f). Immune infiltrates map to the periepithelial cells identified by distance-to-epithelium analysis (Fig. 5a) and corroborate the concept of two dominant patterns of immune organization in the breast. Consistent with our analysis of cell densities, we found depletion for eight neighborhoods in older tissues (Fig. 6g,h), including the basal layer neighborhood which contains both myoepithelial cells and basal cells. The most dramatic depletion was for the lobule-enriched neighborhood, characterized by proliferative epithelium. Overall, we describe higher-order multicellular neighborhoods in the breast and find that the density of most neighborhoods is markedly reduced in older tissues concordant with our finding that breast cellularity decreases with age.

### Age-related remodeling of breast tissue architecture

**Fig. 7:**
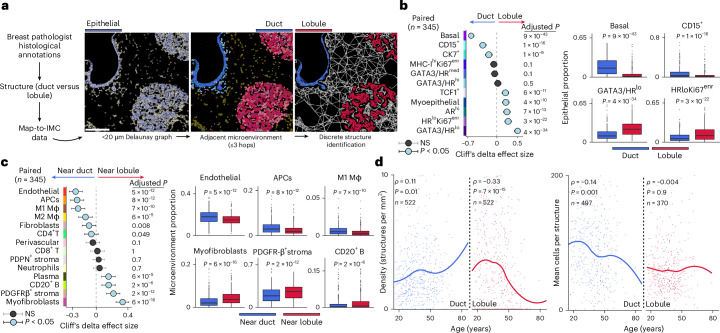
Aging is associated with breast tissue-level remodeling of epithelial structures. **a**, A schematic of epithelial cell classification; scale bar, 150 µm. **b**, Effect sizes for comparisons between epithelial proportion and structure compartment (duct or lobule): circles represent point estimates (median paired difference) and whiskers indicate 95% confidence intervals. The depicted *P* values are derived from two-sided paired Wilcoxon signed-rank tests and are corrected for multiple testing with the Benjamini–Hochberg method. Right: the boxplots depict epithelial proportions for selected phenotypes; the points beyond whiskers are outliers. **c**, Same as **b** for microenvironment cell proportions. For **b** and **c**, the boxes show 25th, 50th and 75th centiles; the whiskers indicate 25th/75th centiles ± 1.5× interquartile range. **d**, Duct and lobule density and cells per structure as functions of age. Correlation coefficients (*ρ*) and *P* values are derived from two-sided Spearman rank correlation coefficient tests; loess regression lines are shown

Early steps of breast carcinogenesis could be affected by local microanatomy, such as the proximity of large ducts or vessels. We therefore meticulously characterized tissue microanatomy and investigated its age-related changes. We annotated images to distinguish ducts from lobules at single-cell resolution (Fig. 7a). Using these annotations, we conducted a paired comparative analysis of the cellular composition of ducts versus lobules. Three epithelial cell phenotypes were enriched in ducts: basal, CD15^+^ and CK7^+^. Epithelial phenotypes enriched in lobules included GATA3/HR^lo^, HR^lo^Ki67^enr^ and AR^hi^ (Fig. 7b). In total, eight of the eleven epithelial phenotypes we identified were differentially enriched between ducts and lobules. To compare the local TME of ducts and lobules, we associated proximate TME cells to adjacent structures by generating spatial graphs (cells within 20 µm were connected) and associating TME cells within three hops of an epithelial cell (Fig. 7a). Comparison of local TMEs revealed ducts were enriched for endothelial cells, macrophages, APCs, fibroblasts and CD4^+^ T cells. Lobules were enriched for myofibroblasts, PDGFRβ stroma and B and plasma cells (Fig. 7c). Together, these findings reveal ducts and lobules as fundamental structural units of the breast and reveal specialized regional stroma and the presence of localized leukocytes.

**Extended Data Fig. 8:**
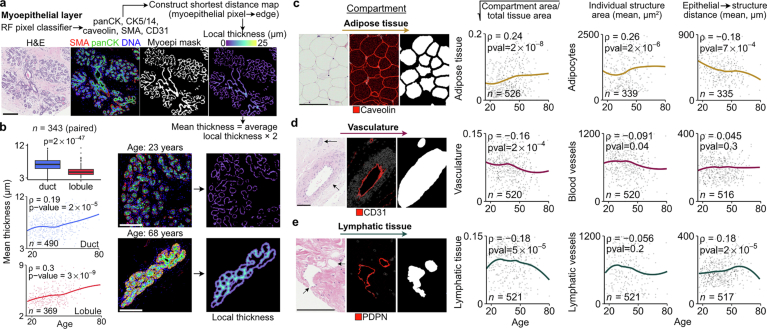
Aging is associated with breast tissue-level remodelling of stromal structures. **a**, Schematic of myoepithelial layer identification. Myoepithelial pixel to edge refers to shortest distance from each white pixel (myoepithelial pixel) to any black pixel (non-myoepithelial pixel). **b**, Relationship between thickness and age for the myoepithelial layer surrounding ducts vs. lobules; p-value above boxplot is derived from a paired Wilcoxon signed-rank test. Representative example images of myoepithelium from young and old patients are shown. **c**, Identification of adipose tissue: in order are the H&E stain, raw marker channel, and final mask. Scatterplots display adipose tissue area as a proportion of total area, mean individual adipocyte area, and the mean shortest distance from epithelial cells to the mask as functions of age. **d**, **e**, As for **c**, for vascular and lymphatic compartments. For **b**–**e**, correlation coefficients (ρ) and p-values are derived from two-sided Spearman rank correlation coefficient tests; loess regression lines are shown. For **b**, boxes show 25th, 50th, and 75th centiles; whiskers indicate 25th/75th centiles ± 1.5 × interquartile range. All scale bars, 150 µm. RF, random forest.

We next investigated the structural dynamics of aging breast tissue by quantifying the density of spatially discrete epithelial structures (ducts or lobules) by age (Fig. 7d). This showed a striking reduction in lobule density (*ρ* = −0.33; log_2_-transformed fold change of −1.96) and a concurrent increase in duct density (*ρ* = 0.11; log_2_-transformed fold change of 0.09). These changes were nonlinear, with a step change around age 50, corresponding to the dramatic estrogen withdrawal associated with menopause. We found that the average number of cells per duct structure decreased slightly with age (*ρ* = −0.14) but that there was no corresponding change for lobules. Ducts and lobules are lined by a layer of specialized myoepithelial cells that rest on the basement membrane. This layer constrains neoplastic proliferations, and its disruption has been associated with nonprogressing in situ carcinoma^27^. We therefore quantified myoepithelial layer thickness using pixel classification and related myoepithelial pixels to the nearest duct or lobule (Extended Data Fig. 8a,b). The myoepithelial layer was thicker in ducts versus lobules. Despite this, its average thickness increased in both ducts (*ρ* = 0.19) and lobules (*ρ* = 0.3) as tissue aged.

**Extended Data Fig. 9:**
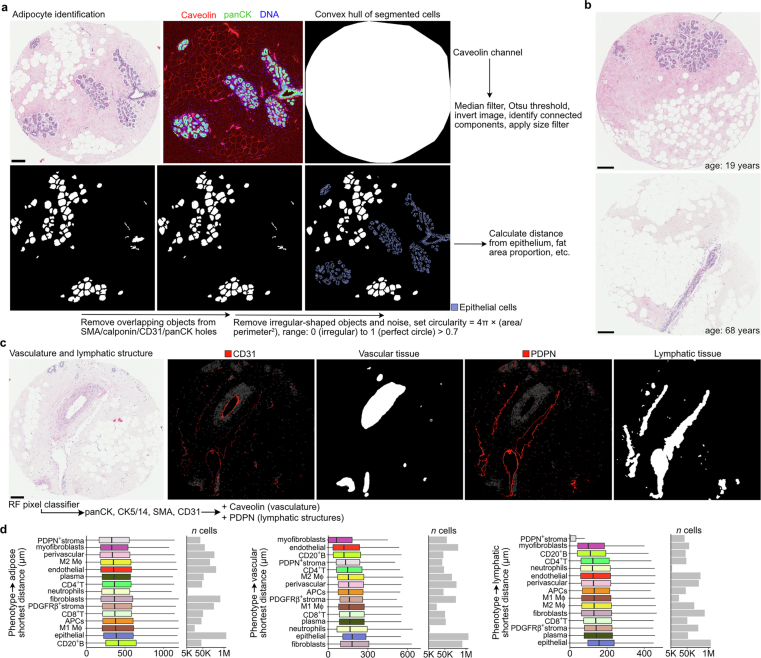
Identification of adipocytes, vasculature, and lymphatic tissue. **a**, Schematic of adipocyte identification. **b**, Representative example images of adipocytes from young and old patients. Representative images are shown from patients selected from the cohort; similar adipocyte morphology and segmentation performance were observed across all patients included in the analysis. **c**, Schematic of vascular and lymphatic structure identification; representative examples are shown from patients selected from the cohort, with consistent segmentation performance observed across all analysed images. **d**, Boxplots depicting shortest distances from individual microenvironment phenotype cells to adipose, vascular, or lymphatic tissue masks. Each data point represents a single cell (technical measurement) pooled across all images and patients. Right-sided bar charts indicate total numbers of cells included per phenotype-structure pair. These analyses are descriptive only and were not used for statistical inference. Boxes show 25th, 50th, and 75th centiles; whiskers indicate 25th/75th centiles ± 1.5 × interquartile range. All scale bars, 150 µm. RF, random forest.

We also mapped the structural dynamics of nonepithelial tissue by quantifying fat and vessels. We used a combination of morphological image processing and random-forest-based pixel classification to quantify and characterize individual adipocytes, blood and lymphatic vessels (Extended Data Figs. 8c–e and 9a–c). Macrophages and stromal cells tended to be close to adipocytes, whereas B cells were most distant. The proportion of tissue area occupied by adipocytes increased with age (*ρ* = 0.24), adipocytes increased in size (*ρ* = 0.26), and the distance to epithelium correspondingly decreased (*ρ* = −0.18). In contrast to adipocytes, B cells tended to be close to blood and lymphatic vessels (Extended Data Fig. 9d). Although their average size remained stable, the proportion of tissue area occupied by vessels decreased with age for both blood (*ρ* = −0.16) and lymphatic vessels (*ρ* = −0.18).

**Extended Data Fig. 10:**
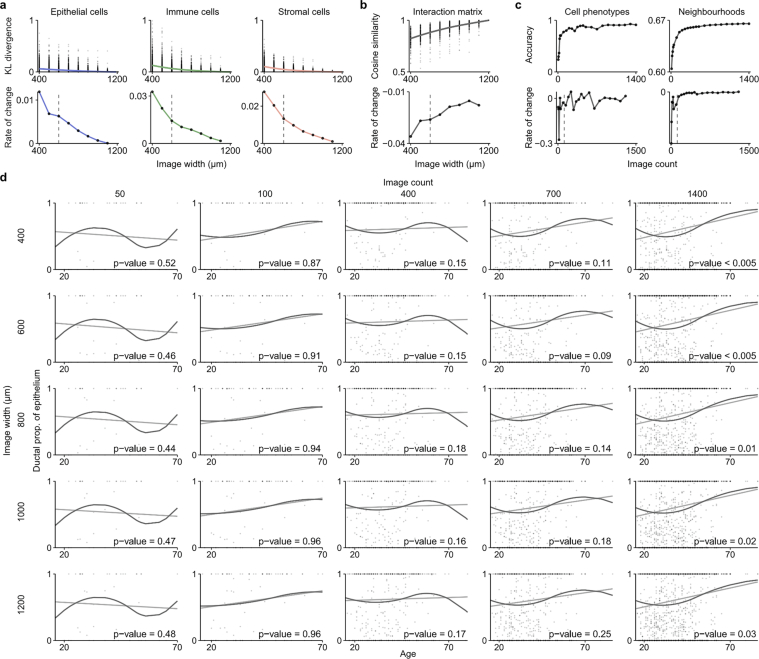
Spatial sampling sensitivity testing. **a**, Sensitivity test of imaging area to the identification of epithelial, immune, and stromal cell distributions. Plotted are KL divergences between cell type distributions of a 1200 µm × 1200 µm image and down sampled images ranging from widths of 400-1200 µm. **b**, Sensitivity test of imaging area to the interaction affinity between cell types as in **a**, with cosine similarities plotted. **c**, Sensitivity tests of number of images to the identification of cell phenotype and neighbourhood distributions. Linear regression curves depict prediction accuracies of classifying phenotypes and neighbourhoods with training sets varying from 5 to 1400 images. **d**, Sensitivity test of age effect to image area and number of images. Plotted are ductal proportions of the epithelium as a function of age, with both quadratic spline curve and linear regression lines shown. Goodness of fit is indicated by p-values assessed from log likelihood ratio tests. KL, Kullback–Leibler.

Given the limited tissue area that could be feasibly imaged, we also conducted sensitivity analyses to clarify the effect of sampling area on representation of tissue architecture. We systematically reduced image area and explored resulting variation in cell type composition, cell–cell interactions and tissue structure. Our findings suggest images greater than 600 µm are robust representations of these features in our dataset (Extended Data Fig. 10). We further assessed the effect of imaging area and imaging sample size on associations between age and tissue structure. We calculated the proportion of cells in the ductal relative to the lobular compartment under 25 combinations of imaging area and imaging sample sizes. We fit a quadratic spline model with a single knot at menopausal age (50 years) and a linear model to the data. The log-likelihood ratio test comparing the two models showed that at an (image) sample size of 1,400 we can observe statistically significant goodness of fit, illustrating that the spatial architecture of the normal breast can be robustly represented when given a large enough sample size at 1,200 (Extended Data Fig. 10c,d). Overall, these results show that there is a trade-off between sampling intensity and sample size but that our dataset was well powered for evaluating the relationship between tissue structure and age.

A small proportion of tissues (79 out of 1,494 images) contained benign changes such as secretory change and apocrine metaplasia; we annotated images for these changes at single-cell resolution (Supplementary Fig. 8). Secretory cell change occurred in three instances (all under 50 years) and was characterized by expression of CD15 both in secretions and the apical surface of epithelial cells (Supplementary Fig. 8c). Apocrine metaplasia showed a characteristic expression profile lacking ER and PR but showing high expression of AR (retaining FOXA1 and showing apical CD15 and patchy HER2) (Supplementary Fig. 8d). These findings are illustrative of the phenotypic transdifferentiation dynamics associated with breast aging and support the fidelity of these highly curated data.

In summary, our quantitative analysis of tissue structure revealed a marked depletion of lobules around the age of menopause accompanied by a steady increase in adipose tissue and reduction in vessels. We conclude that age-related remodeling of breast architecture is dramatic and spans both epithelial and nonepithelial structures.

### Tissue aging in the adult breast is dominated by menopause

**Fig. 8:**
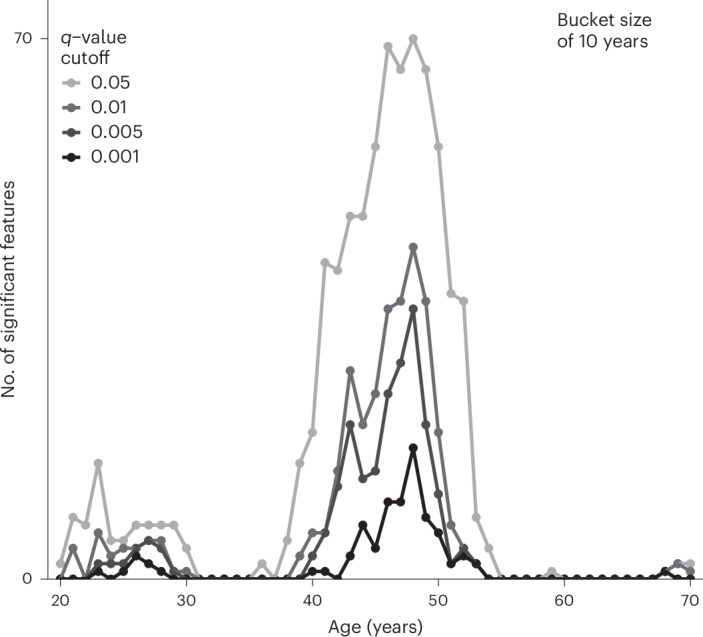
Nonlinear patterns in breast tissue aging. Nonlinear sliding window analysis demonstrating the number of significant features out of 233 as a function of age, with different *q*-value cutoffs displayed and a window size of 10 years.

Finding that tissue architecture altered dramatically around menopause prompted us to ask whether the breast is subject to nonlinear aging at one or several periods during the lifespan. Notably, recent work based on plasma molecules^28,29^ and the microbiome^29^ revealed that age-related molecular dynamics are highly nonlinear with several phases of acceleration. It is not, however, known whether comparable waves of accelerated change also occur at the level of tissue phenotype. We therefore employed a data-driven approach to determine whether breast tissue aging is also characterized by similar dynamics. We explored associations with all the multiscale tissue phenotypes we had defined (233 in total spanning pixel, cell and architectural features) (Methods, Supplementary Table 5 and Supplementary Fig. 9) and age using sliding-windows to identify the ages at which they are most subject to change (where most associations were statistically detectable). We found that breast tissue, unlike circulating plasma molecules, is dominated by a single aging peak in the late 40 s (Fig. 8) corresponding to menopause. A much smaller second peak was also evident in the 20 s, which included B and plasma cell density, but this was dwarfed by the menopausal peak in the late 40 s. These findings indicate that adult breast tissue aging is predominantly menopause-related and suggest that hormone-sensitive tissues may not conform to a model of recurrent phases of change.

## Discussion

Hormonal factors and aging itself cumulatively remodel breast tissue. We precisely characterized this process to better understand its implications for the development of cancer. Using spatial proteomics, we quantified the composition, cellular interactions and tissue structure of normal breast tissue from over 500 women spanning a range of ages.

We find that the breast undergoes general age-related decline: it becomes less cellular and contains fewer proliferating cells across cell types. There is a notable change in the spatial context of epithelial cells too; they are characterized by fewer heterotypic interactions (with immune and stromal cells) in older tissues. The aged TME favors inflammation with a changed composition enriched for M2 macrophages and CD8^+^GZMB^+^ T cells. Tissue architecture is restructured with dramatic depletion of lobules around the age of menopause, increased fat and reduced vasculature. The age-incidence of cancer is largely attributed to steady accumulation of somatic mutations^30^, but our data suggest that the tissue ecosystem is in general more permissive of carcinogenesis with age^31^. Recent studies have shown, for example, that luminal breast epithelial cells accumulate somatic mutations^2^ and copy number alterations^6,32^ akin to those found in primary tumors. It is plausible that some cells bearing these alterations evoke an immune response through diverse mechanisms such as expression of neoepitopes^33^ or, in the case of aneuploidy, cytosolic sensing of DNA^34^. Some of our findings, such as reduced immune cell density, fewer epithelial-immune interactions in older tissues and increased immunosuppressive M2 macrophages, suggest that the potency of these responses may wane with age creating a microenvironment more permissive of carcinogenesis.

Although breast tissue is not routinely sampled in the clinic, the breast is regularly assessed by screening mammography and mammographic density has emerged as a robust risk factor for developing breast cancer^24^. The known reduction in mammographic density with age is thought to correspond to involution of breast tissue^35^. Our findings corroborate this age-related decline in breast cellularity and show it extends to stromal and immune cells. Women with high mammographic density for their age are at elevated risk of breast cancer^36^, highlighting that the rate of age-related decline we observe probably differs between women and probably affects their cancer risk. These differences may be partly explained by the impact of hormonal levels on epithelial cell proliferation though it remains unclear how this effect is modulated by the microenvironment.

How the dynamics of the aging microenvironment influence tumor progression is an active area of research^37^. Our comparative analysis of the composition of the breast microenvironment revealed B cells as an outlier in the extent of their enrichment in younger tissues. Using single-cell transcriptomics, others have also found that memory-switched B cells characterize younger (premenopausal) breast tissue^10^. The role of B cells in the normal breast is probably multifactorial, but we speculate that their marked enrichment in the young may be related to the need for secretion of immunoglobulins as part of the early stages of milk production, known to contain B cells^38^ and high levels of IgA needed to coat the gut lining of the newborn^39^. Nonetheless, both CD8^+^ T cells and APCs (including dendritic cells) were also more prevalent in younger tissues. We speculate that immune surveillance by resident leukocytes wanes with age, and the immune response is consequently a less effective extrinsic tumor suppressor in aged tissues. This is compounded by an inflammatory shift that includes increased M2 macrophages, which have been implicated in age-related tumor progression^37,40^.

Past attempts to identify age-related changes in the breast have used both animal models and human tissues^41–45^. One study used mouse models and single-cell transcriptomics to isolate the impact of age on mammary glands^45^. By comparing young and aged tissues while controlling for confounders, this study revealed changes among both epithelial and microenvironment cells. One subset of macrophages characterized by expression of matrix metalloproteinases increased with age, whereas CD163^+^ macrophages decreased. This is contrasted by our finding of an overall decrease in macrophage density, but an increase in the proportion of the microenvironment comprising M2 (CD163^+^) macrophages. A second murine study, however, found that M2-like macrophages and *Gzmk*^+^ T cells increased with age^43^, in agreement with our observations. These different findings are probably due to distinct study designs, assays and methods of analysis. Other work that is concordant with our findings includes an IHC study of normal breast tissues from over 100 women which found that the density of lymphocytes (B cells and T cells) decreases with age but that M2 macrophages increase^46^. Our single-cell spatial analysis of a large human tissue collection shows that aging appears to remodel the breast across multiple scales. Older tissue contains fewer and different cells, in altered activation states with changed morphology. Our data support the known decrease in immune function with age^47^ but suggest this occurs in the context of a more general decline that underlies organ-level remodeling.

Past work has suggested that luminal progenitors with a bias toward basal differentiation accumulate with age^48^ and, further, that luminal epithelial cells are the key site of integration of aging-related changes in proximate tissue^49^. Of the epithelial phenotypes we defined, only basal cells showed a substantial increase in older tissues. Since this phenotype was highly enriched in duct regions, some of this shift is explained by the reduction in lobules and relative increase in ducts we observed in older tissues. After adjustment for the proportion of epithelial cells located in a duct or lobule, however, basal cells remained significantly enriched in older tissues in agreement with published research^48^ (Extended Data Fig. 4f) and supporting the idea that increased basal cells in older tissues are partly explained by loss of lineage fidelity. Indeed, we have previously noted strong age-related correlations of EZH2 and H3K27me3 expression in these tissues^5^.

Recent multiomic studies of circulating molecules have shown that aging is characterized by highly nonlinear dynamics^28,29^. One study, for example, revealed two peaks of change in molecular aging markers at 44 and 60 years^29^. However, whether similar dynamics characterize tissue aging is not known. To address this in breast tissue, we applied similar analyses to our dataset. Unlike circulating molecules, we found a single dominant peak in the late 40s, which coincides with menopause. This finding, based on a data-driven methodology emphasizes the importance of hormones to breast tissue aging. Although menopause appears to dominate changes in tissue phenotype, however, this is contrasted by somatic mutations that are known to accumulate steadily with chronological age^2^ consistent with other organs.

As the spatial omics revolution unfolds, how best to interpret complex cancer tissue images is an increasingly urgent question. By precisely enumerating tissue resident cells and their interactions in normal breast tissue, our extensively annotated dataset will serve as a reference atlas to facilitate interpretation of spatial breast cancer data as well as studies of breast biology. A limitation of our data, however, is that linked metadata are limited to age. Other factors, especially those impacting hormonal levels, will affect breast tissue, but those data were not available for our analysis. In addition, although we lack data on ethnicity, our collection is also likely to under-represent minorities. Further, by using a targeted proteomic analysis we have probably overlooked some relevant tissue features. Another limitation is that we did not compare tissue profiles between women at high-risk of breast cancer, such as those bearing pathogenic variants in *BRCA1* and *BRCA2*, to the background population. Recent work suggests that histologically normal tissue in *BRCA1* and *BRCA2* carriers is characterized by features of accelerated aging such as loss of lineage fidelity^50^ and immune exhaustion^11^. Future multiplexed imaging studies of high-risk tissues are warranted to characterize the role played by spatial context in mediating these differences. A further limitation of our data is that it was derived from a small proportion of the tissue in the breast and that its exact anatomic location within the organ was not known. Similarly, we have relied on two-dimensional images to quantify cells and their spatial relationships, which may differ in three-dimensions^51^. Nevertheless, our approach enabled a large-scale analysis to precisely quantify variation with age and across individuals. Our multiscale atlas reveals extensive remodeling of breast tissue illustrative of general age-related tissue involution. The spatial context of breast epithelial cells changes as tissues age and this may affect the likelihood of tumor initiation.

## Methods

### Study design, TMA construction and metadata

The normal breast dataset was collected at the British Columbia Cancer Research Institute, part of the Provincial Health Services Authority, with ethics committee approval. Patient specimens were obtained from the BC Cancer/University of British Columbia Research Ethics Board protocol titled ‘BC Cancer Mammary Cell Bank’ under ethics approval H19-03798, which provides for informed consent or waiver of consent for secondary research use of anonymized legacy biobank human tissues surplus to diagnostic requirement. Tissue analysis with these specimens was conducted under protocols H25-01125 and H19-03794^5^. The TMA consists of formalin-fixed paraffin-embedded samples of normal breast tissue from 537 cancer-free women who underwent reduction mammoplasty surgery at various hospitals in Vancouver. The dataset includes information on the age of patients at the time of surgery, with a median age of 38 years (range 15–86 years). Up to five, 1.5-mm cores were taken from representative areas of breast epithelium and randomized across and within slides in the TMA. Two sequential sections of FFPE tissue were prepared from the TMA blocks. One section was stained with hematoxylin and eosin (H&E) using an automated staining system (Leica ST5020 Stainer/TS5025 Transfer Station/CV5030 Coverslipper Workstation). The H&E slides were scanned with the Leica Aperio AT2 Automated Digital Whole Slide Scanner and dearrayed for analysis. The adjacent section was processed with IMC as described below. Ultimately, 527 patients were included in subsequent analyses.

### Antibody panel design, metal conjugation and data acquisition

Antibody panel design (including antibody descriptions, concentrations, clone information and metal isotope tags used) is provided as Supplementary Table 1, with rationales^52^ for each marker presented in Supplementary Table 2. Antibody staining patterns and concentrations, including validation of intensity, specificity and signal-to-noise ratio, were evaluated by inspection of immunofluorescence and IMC images from a variety of control tissues, including tonsil and breast. Antibody–metal conjugations and tissue labeling were completed as previously described^53–55^, including conjugation of platinum^56^ and bismuth^57,58^ isotopes. Finally, stained tissues were ablated with a Hyperion+ Imaging Mass Cytometer (Standard BioTools), and intensities were quantified using time-of-flight mass spectrometry^14^.

### Image processing and single-cell measurements

The full workflow is described by Extended Data Fig. 1. Raw text files were converted to multistack image tiff files using the imctools Python package^59^. Then, single-cell segmentation was performed with the deep-learning model DeepCell Mesmer^23^ by passing a combination of iridium and histone H3 for nuclear masks and a combination of CK5/14, panCK, CD45, HLA-ABC, HLA-DR and SMA for cell masks. Before taking measurements, multistack tiffs were filtered for single hot pixels and single-cell proteomic measurements were taken by computing the mean ion count for each segmented cell using CellProfiler version 4.0.6^60^.

Spillover compensation was performed by ablating dried drops of metal-conjugated antibody pipetted onto an agarose-coated glass slide. A spillover matrix quantifying spillover crosstalk was computed as previously described^55^ using the R Bioconductor CATALYST^61^ package and subsequently used to correct single-cell measurements using the imcplugins package version 4.2.1 in CellProfiler^59,60,62^.

### IHC validation

IHC measurements on the normal breast TMA were used to examine the correlation of protein expression between IMC and IHC. For each IHC image, the percentage of positively stained cells for protein markers were previously annotated by breast pathologists at the BC Cancer Research Institute/University of British Columbia^5^. Spearman rank correlations between the mean expression intensity in IMC and the percentage of positive cells in IHC were computed for common proteins shared between the two modalities: CK5, Ki67, FOXA1, ER, PR and HER2.

### Cell phenotyping and thresholding

Single cells were classified as epithelial or microenvironment cells using an ensemble of in-house deep-learning models with modified Mesmer^23^-backbone applied to preprocessed images (clipped at the 99th centile and minimum–maximum scaled) on a subset of channels (Ki67, HLA-DR, H3, CK5/14, CD45, panCK, DNA1, CD4 and CD8). Pixel-level predictions were aggregated via single-cell masks before validation by visual inspection of tissue morphology and cytokeratin expression. A semi-supervised approach was then taken to further characterize epithelial and microenvironment phenotypes in their respective compartments, shown in Extended Data Fig. 1. Cellular expression values across the entire dataset were scaled and clipped at the 99th centile, and then 200,000 randomly sampled cells from each compartment were clustered with the PhenoGraph algorithm^63^ (*k* = 590) to identify distinct phenotypes. Resulting clusters were mapped back to single cells, and heatmaps of *z*-scored median expression values were inspected. Similar clusters based on expression values and visual cellular inspection were merged to obtain the final set of epithelial and microenvironment clusters. Then, a random forest classifier was trained based on single-cell expression and cluster assignments and was used to predict the phenotypes for the rest of the dataset. Final cluster validity was investigated by inspecting images annotated with cluster labels and expression profiles to ensure cell morphology and expression values were concordant with the cluster label (see Supplementary Figs. 1 and 2 for representative phenotype images). Lastly, segmented objects with areas less than 10 µm^2^ or greater than 250 µm^2^ were excluded, and images containing fewer than 100 segmented cells were removed from further analyses.

Marker positivity for Ki67 and luminal markers (FOXA1, GATA3, ER, AR, PR and HER2) was determined using pixel-based *k*-means clustering to identify contiguous areas of positive and negative pixels. These areas were then mapped to single cells, returning the number of positive pixels normalized to cell size for each cell. Thresholds of these values were determined by visual inspection (separately for each marker due to varying signal-to-noise ratios) using a randomly selected set of 20 images to validate each threshold (see Extended Data Fig. 2 for representative thresholding). The same method was used to perform thresholding for natural killer and T cell markers (CD45, CD56, PD-1, TCF1 and GZMB).

### Differential phenotype density and positive fraction abundance analysis

For each patient, the proportion and density of phenotypes were computed separately by epithelial and microenvironment compartments, and the fractions of proliferating cells (Ki67^+^) were computed for each phenotype. Tissue areas for density calculations were quantified by fitting convex hulls around all segmented cells as tissue did not cover the entirety of all ROIs. Patients were segregated based on an age cut-off of 50 years, and median densities for each phenotype were compared between age groups with Mann–Whitney *U* tests. All *P* values were adjusted for multiple testing by the Benjamini–Hochberg method, and corresponding Cliff’s delta effect sizes (including error estimates) were calculated with the R effsize package^64^. Continuous associations with age were evaluated with Spearman’s rank correlation coefficient tests.

Differences in the cellular phenotypic composition between age groups were tested using trials–events binomial generalized linear mixed-effects models. Each observation in these models corresponded to an image and was weighted by the total number of cells (to account for differences in the precision of proportion estimates). Both image and patient identifiers were modeled as grouping random effects terms to account for the hierarchical structure of the data. To determine whether age-related compositional differences among cellular phenotypes could be explained by the balance between duct/lobule structures, models were also fit with a term representing the proportion of epithelial cells in an image that were ductal (where epithelial cells can only belong to two categories: lobular or ductal); this term was square root transformed. Hypothesis testing was conducted using the glht function from the multcomp R package.

### Cell morphology

We assessed cell morphology by taking single-cell and single-nucleus measurements of morphological parameters with CellProfiler using the cell and nucleus masks obtained from Mesmer segmentation. Differences in average cell areas and major:minor axis ratios between age groups were quantified with Mann–Whitney *U* tests for each patient.

### Spatial distribution and neighborhood analysis

Spatial statistics and nearest distances to each epithelial cell were computed by representing distributions as point pattern objects with the R spatstat package^65^. Bivariate Ripley’s *K* analysis was used to determine clustering tendencies of microenvironment phenotypes around epithelial cells with *K*_cross_ function calculations. For each image, 199 Monte Carlo simulations of complete spatial randomness (CSR, cells distributed completely randomly within imaged tissue area) were performed to assess significance (*P* < 0.01) with a two-tailed test. Then, for each phenotype, the proportion of images in which the observed distribution clustered above and/or below simulated CSR distributions was computed and reported. Distance to epithelium changes with age were assessed with Mann–Whitney *U* tests (with Benjamini–Hochberg corrections to *P* values), and the corresponding Cliff’s delta effect sizes and error estimates were calculated with the R effsize package.

Cell–cell interaction quantification was estimated by constructing Delaunay graphs of cells and removing edges greater than 20 µm. Then, interactions were counted and normalized by dividing interaction counts by the number of cells in the epithelial and/or microenvironment compartments, depending on the interaction type being considered (epithelial–epithelial, epithelial–TME or TME–TME).

Recurrent multicellular neighborhoods were characterized with the CellCharter framework^25,26^, which utilizes a cell expression matrix and spatial networks to predict spatial community clusters based on neighbor similarity. First, the entire raw phenotypic dataset was scaled and clipped at the 99th centile to prepare the phenotypic matrix. Then, using the corresponding cell centroids, the Squidpy library^66^ was used to perform Delaunay triangulation to connect neighbors with edges. After clipping the 99th centile edges based on the longest distances per image, Gaussian mixture models (GMMs) were fit based on feature similarities between three-step neighbors. Since GMMs require the number of clusters as a user-defined parameter, we performed repeated training of GMMs for a range of 4 to 15 clusters and for each cluster number computed the Fowlkes–Mallows index for cluster stability based on five repetitions. The decision to select *n* = 10 clusters was done by identifying locally stable Fowlkes–Mallows index solutions, validating the cluster assignments by looking at images (see Extended Data Figs. 6 and 7 for representative spatial clusters) and using a cluster number we might expect based on the fidelity of our data (number of markers and expected biological/spatial diversity). Lastly, we used the selected GMM to predict final spatial cluster assignments that were mapped back to cell and phenotype assignments. Spatial clusters were labeled by considering both the enrichment of phenotypes and the physical organization of spatial cluster-assigned cells.

Spatial communities were characterized by constructing full Delaunay graphs for each image, using the igraph library^67^ to create subgraphs for cells with the same spatial cluster assignments. Communities were subsequently identified as connected components of subgraphs, and all communities with fewer than ten cells were excluded from further analyses to remove noise. The Shannon diversity by cell phenotype of each community was computed, along with the mean vertex degree (number of cell–cell contacts) to distinguish each community type. Community density refers to the number of communities per unit area.

### Breast epithelial histological analysis

Extensive image annotation was conducted under the supervision of a breast pathologist (H.R.A.) to identify duct/lobule regions on adjacent H&E sections. Corresponding epithelial cells from the IMC dataset were labeled accordingly as either ductal or lobular, and the surrounding microenvironment cells were identified as the microenvironment cells within three hops of an epithelial cell in a 20-µm distance-constrained Delaunay graph. Only those patients with both ductal and lobular regions acquired were included in abundance analyses (*n* = 345), and paired Wilcoxon signed-rank tests were used to quantify differences in abundances of epithelial and microenvironment phenotypes in ductal versus lobular regions. To distinguish distinct duct/lobule structures, a community-based approach was taken, constructing full Delaunay graphs and creating subgraphs for cells with the same duct/lobule/microenvironment assignment. Structures were subsequently identified as connected components of subgraphs, and the abundance and number of cells of duct/lobule structures was characterized. Only patients with ducts and/or lobules are included in the calculations of cells per duct/lobule structure.

In addition, histologically benign epithelial cell changes were annotated under the supervision of a pathologist (H.R.A.), identifying five broad categories present in the normal breast dataset: secretory cell change, fibrocystic change, apocrine metaplasia, columnar cell change and usual ductal hyperplasia. After epithelial cells were labeled with these annotations, a similar graph-based approach was used to identify surrounding microenvironment cells. Due to limited sample sizes, rigorous statistical quantification and comparisons could not be performed, but phenotype enrichments in histological cell change categories were computed and reported as the ratio of observed counts to expected counts. Expected numbers of cells in this calculation were derived from the proportional distribution of cell types observed in the total dataset.

### Characterization of nonepithelial tissue structure

All tissue structure identifications (myoepithelial layer, adipose, vascular and lymphatic tissue) are illustrated in Fig. 7 and Extended Data Fig. 8. The myoepithelial layer was characterized by training a random forest pixel classifier (Ilastik^68^) on channels panCK, CK5/14, caveolin, SMA and CD31 to generate binary myoepithelial layer masks. Then, distance maps calculating shortest distances from each myoepithelial pixel to any nonmyoepithelial pixel were created using the EBImage R package^69^, multiplying the resulting distance values by two as a proxy for local thickness. To distinguish ductal versus lobular myoepithelial layer thickness values, each myoepithelial layer pixel was classified based on its proximity to a ductal/lobular epithelial cell, and then separate local thickness averages were taken.

Adipose tissue was identified from raw IMC image data. For identifying adipocytes, the caveolin channel was clipped at the 99th centile, median filtered, binarized with the Otsu method and inverted, resulting in objects from connected components. Because caveolin often also surrounds epithelial areas, the objects identified from using the panCK channel were subtracted from overlapping caveolin channel objects. The resulting adipocytes were constrained at a minimum object size of 300 µm^2^ and a circularity = 4π(area/perimeter^2^) ≥ 0.7.

Vascular and lymphatic masks were generated from random forest pixel classifier training with panCK, CK5/14, SMA, CD31 and caveolin for vasculature or podoplanin for lymphatic structures. Resulting binary masks had any holes filled in and a minimum object size threshold of 10 µm^2^. Distances from cells to masks, mask proportion of total tissue area and number of vessels were quantified.

### Core-to-core variability analysis

Cellular counts for both epithelial and microenvironmental phenotypes were aggregated per core and normalized to obtain proportional cell type distributions. Within each patient, pairwise Pearson correlations were computed on these proportions to assess compositional similarity across imaging cores, and the variance of these proportions was calculated to capture intra-patient heterogeneity.

### Sampling sensitivity analysis

To compute the sensitivity of our findings on core size and sample size, a fourfold analysis was performed by analyzing cell type distribution, cell type co-occurrence, classification accuracy and age effect on epithelial cells.

Sensitivity tests with respect to core size were based on cell type distributions and cell type co-occurrences. All IMC images were cropped into squares with sizes ranging from 1,200 µm × 1,200 µm to 400 µm × 400 µm, decreasing by 100 µm per square. To emulate the choice of a pathologist’s frame of view when imaging in smaller dimensions, the down sampled images were cropped centered at the position of highest cell density in the original image determined by kernel density estimation. For each down sampled image, its cell type distributions stratified by epithelial, immune and stromal were calculated and an enrichment matrix of cell type adjacencies was computed via a permutation test. The forward Kullback–Leibler divergence between the cell type distribution of each down sampled image and that of the 1,200 µm × 1,200 µm image was computed. Cosine similarities were also calculated between the cell type co-occurrence enrichment of each down sampled image and that of the 1,200 µm × 1,200 µm image. A linear regression model was fit on all metrics across image widths, and the rate of change was computed to estimate sensitivity.

To analyze sensitivity with respect to sample size, a set of annotations at cellular resolution including cell phenotypes and neighborhoods were used. The original set of IMC images were randomly subsampled to 24 sets of images with a total count between 5 and 1,400. The annotations for each set of images were then used to train multiclass gradient boosting classifiers. Performance of each classifier was evaluated on prediction accuracy on the original dataset, and rates of change across image counts were computed to estimate sensitivity.

To compute sensitivity with respect to age effect, the proportion of ductal cells in the epithelial compartment with respect to age was computed for all combinations between five image size widths (400, 600, 800, 1,000 and 1,200 µm) and five image sample sizes (50, 100, 400, 700 and 1,400). A quadradic spline model with a single knot at age of 50 years was fit to the data, along with a linear model to perform a likelihood ratio test for assessing sensitivity.

### Nonlinear sliding window analysis

We utilized the package DEswan^28,29^, a differential expression sliding window analysis, to test for significant differences in feature expression associated with aging. Specifically, cell and tissue features (Supplementary Table 5) analyzed between ages 20 and 70 years were examined using linear models applied within a sliding window of 10 years (±5 years around each central age). A 10-year window was selected because narrower windows (for example, 5 years) lacked statistical power to detect meaningful differences, whereas broader windows would overlook nuanced, nonlinear age-related changes. All *P* values were corrected using the Benjamini–Hochberg method to obtain *q*-values, and robustness was confirmed by showing that changing window sizes or using alternative thresholds produced consistent aging wave locations (Supplementary Fig. 9a), whereas permuting patient ages resulted in an abscence of notable findings (Supplementary Fig. 9b).

### Statistics and reproducibility

All statistical analyses were performed in R (version 4.2) unless otherwise specified. Nonparametric tests were primarily used for group comparisons (Mann–Whitney *U* tests, Wilcoxon signed-rank tests) due to the large sample size and potential deviations from strict normality assumptions. Effect sizes were quantified using Cliff’s delta with associated error estimates where appropriate. Correlation analyses were performed using Spearman’s rank correlation coefficient. Generalized linear mixed-effects models were fit using binomial distributions with logit link functions, incorporating image and patient identifiers as random effects to account for hierarchical data structure. Multiple hypothesis testing corrections were performed using the Benjamini–Hochberg method.

No statistical methods were used to predetermine sample sizes. Cohort sizes exceed those in prior large-scale spatial and breast tissue atlases. Data distributions were assumed to be normal where required for modeling but were not formally tested. Individual data points are shown where appropriate.

As this was an observational study of archived human tissue samples analyzed via IMC, no experimental randomization was applicable. Data collection and analysis were not preformed blind to experimental conditions. No samples or data points were excluded beyond predefined quality control thresholds, including image quality criteria, segmentation integrity, minimum object size filters and minimal cell count requirements per image as described above.

### Reporting summary

Further information on research design is available in the Nature Portfolio Reporting Summary linked to this article.

## Supplementary information

Supplementary Figs. 1–9.

Reporting Summary

Supplementary Tables 1–5.

## Data Availability

All IMC, derived images and processed single-cell data, together with patient age metadata and summary counts of cell phenotypes, can be accessed via Zenodo at 10.5281/zenodo.18418221 (ref. ^70^). Other data generated during the study are available from the authors upon reasonable request.

## Code availability

The pipeline used for image processing from the raw IMC data is hosted via GitHub at https://github.com/BodenmillerGroup/ImcSegmentationPipeline and https://github.com/vanvalenlab/deepcell-tf. All additional analysis code can be accessed via GitHub at https://github.com/HRazaAliLab/IMCBreastAgeingAtlas or via Zenodo at 10.5281/zenodo.18418221 (ref. ^70^).
