# Supplementary material for: Single-cell spatial atlas of the aging human breast
Source: Nat Aging. 2026 Mar 31;6(4):916–31. doi: 10.1038/s43587-026-01104-3 (PMC13099655; doi:10.1038/s43587-026-01104-3)
Supplement: Supplementary file 1 — Supplementary Figs. 1–9. [file 43587_2026_1104_MOESM1_ESM.pdf]

nature aging

Supplementary information https://doi.org/10.1038/s43587-026-01104-3

Single-cell spatial atlas of the aging human breast

In the format provided by the authors and unedited

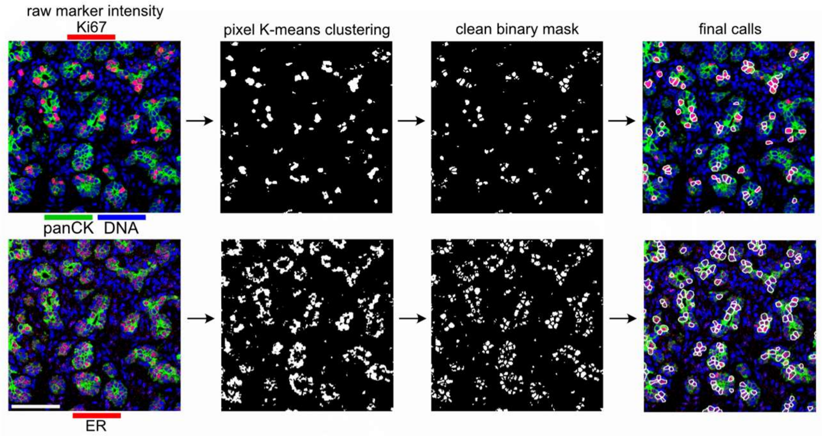

Supplementary Fig. 1|Schematic of positivity/negativity calling with pixel-based *k*-means clustering. Representative images of Ki67 and ER calling are shown. (1) Raw marker intensities, (2) masks after performing k-means clustering placing pixels into two groups (positive or negative), (3) cleaned binary mask after removing small or isolated clusters, filling holes, and smoothing boundaries, and (4) final positive cell calls after manually selecting a threshold of the percentage of pixels in a segmented cell that are positive. The *k*-means threshold is unique to each marker and depends on each marker's signal-to-noise ratio along with its specificity for positive cells. Scale bar, 100 µm.

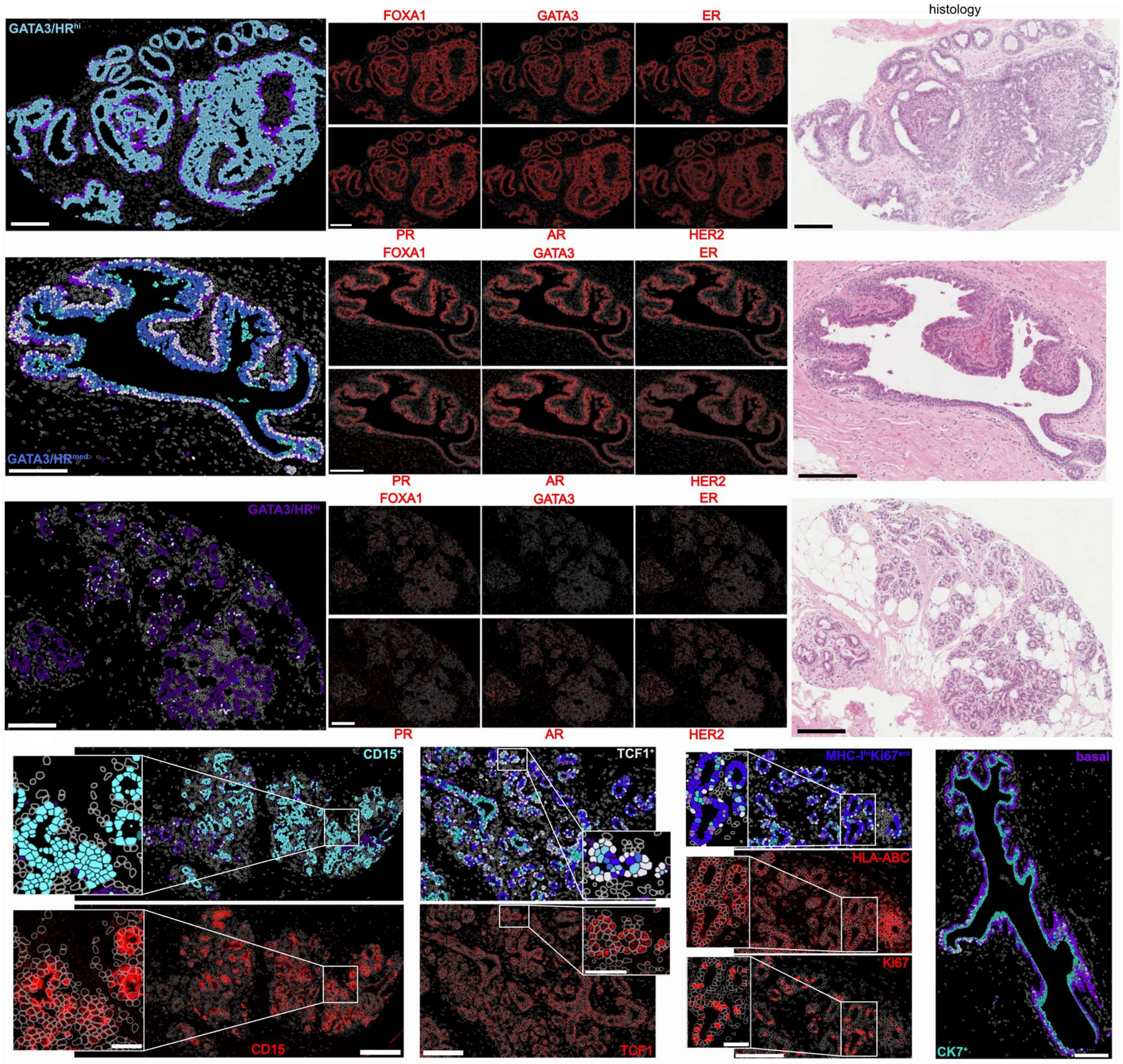

Supplementary Fig. 2|Representative epithelial phenotype examples. Depictions of GATA3/HR$^{lo/med/hi}$, CD15^{+}, TCF1^{+}, MHC-I^{hi}Ki67^{enr}, basal, and CK7^{+} phenotypes. All epithelial cells are coloured; microenvironment cells are non-coloured. Scale bars for full images, 150 µm; scale bars for zoomed insets, 50 µm.

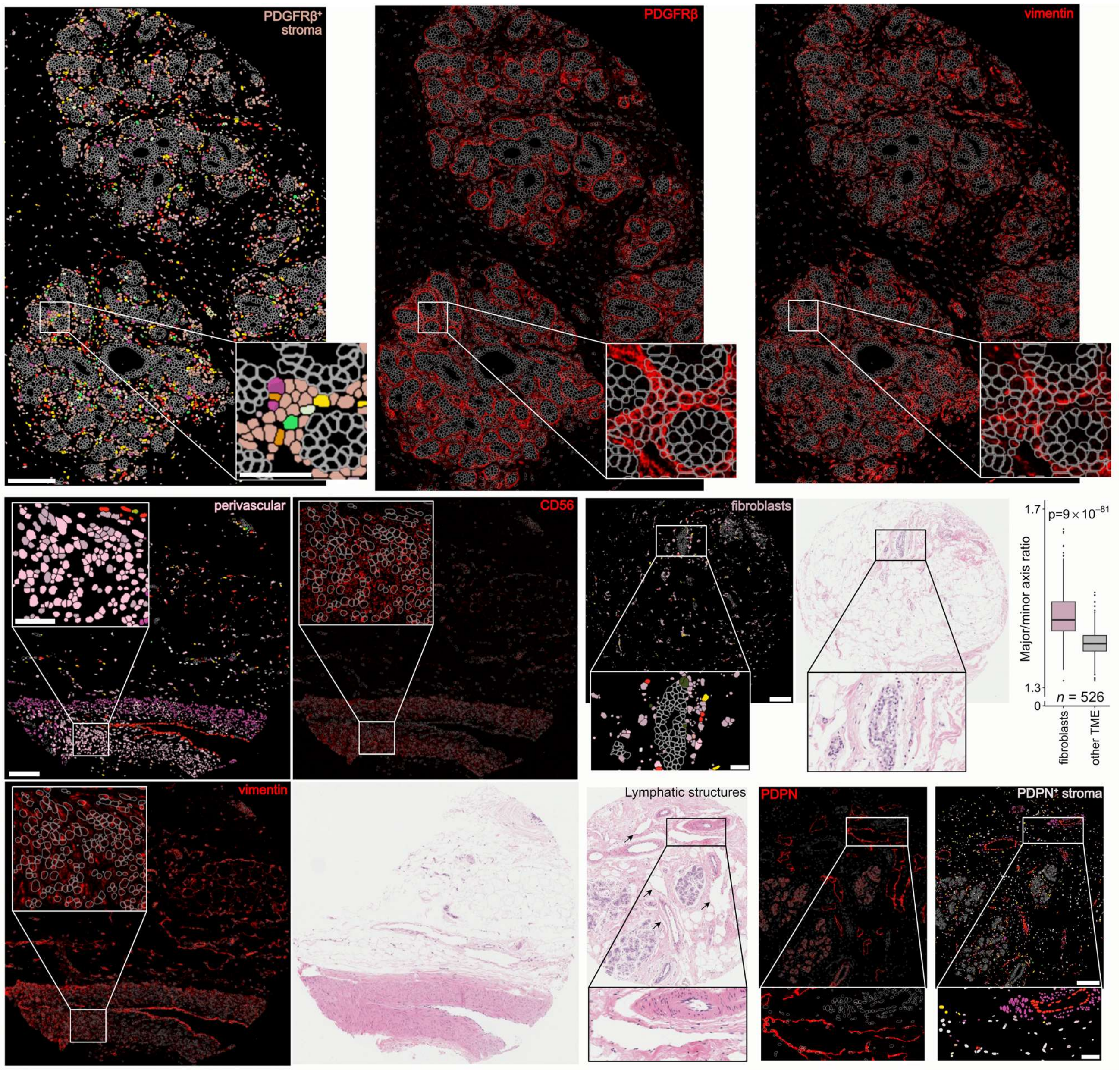

Supplementary Fig. 3|Representative microenvironment phenotype examples. Depictions of PDGFRβ⁺ stroma, perivascular, fibroblasts, and PDPN⁺ stroma phenotypes. Boxplots display mean cellular major-to-minor axis ratios; p-value is derived from a two-sided paired Wilcoxon signed-rank test. All microenvironment cells are coloured; microenvironment cells are non-coloured. Scale bars for full images, 150 µm; scale bars for zoomed insets, 50 µm. TME, tissue microenvironment.

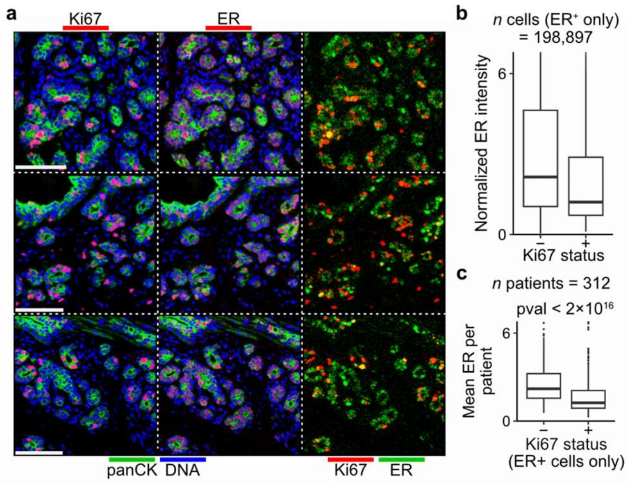

Supplementary Fig. 4|Distributions of ER expression among ER$^+$ cells by Ki67 status. a, Representative regions of epithelium with ER/Ki67 double-positive cells (scale bar, 100 µm). **b**, Boxplot of normalized ER expression (scaled and clipped at the 99th centile) at the cell level in the ER$^+$ compartment. **c**, Paired Wilcox test comparing mean ER expression of ER$^+$Ki67$^+$ vs. ER$^+$Ki67$^-$ cells in patients with both types of cells (*n* = 312).

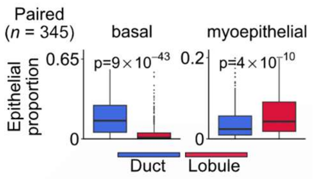

Supplementary Fig. 5|Epithelial duct and lobule proportions for basal and myoepithelial cells. Depicted p-values are derived from two-sided paired Wilcoxon signed-rank tests and are corrected for multiple testing with the Benjamini-Hochberg method. Points beyond whiskers are outliers.

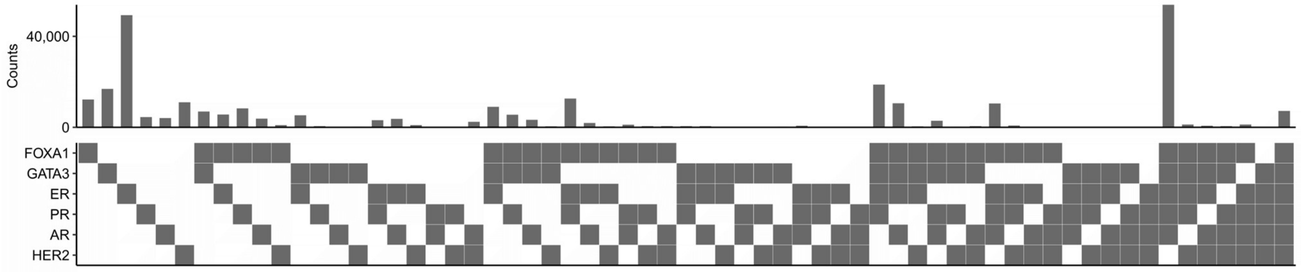

Supplementary Fig. 6|Frequencies of hormone receptor and related protein-expressing marker combinations in the epithelial compartment. All 63 combinations are shown.

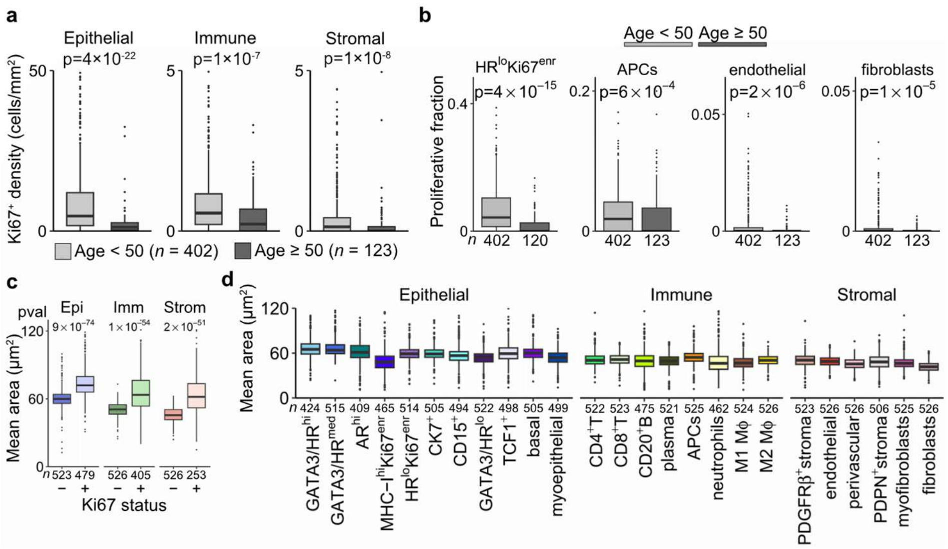

Supplementary Fig. 7|Ageing is associated with breast cell level changes in proliferation and morphology. **a**, Ki67⁺ cell density for epithelial, immune, and stromal phenotypes. P-values are derived from two-sided unpaired Wilcoxon tests comparing age groups within each compartment. **b**, Boxplots of proliferative cell fractions for selected phenotypes from Fig. 4c. **c**, Boxplots of mean cell area for each compartment, split by Ki67 status. Depicted p-values are derived from two-sided unpaired Wilcoxon tests. **d**, Boxplots of phenotype mean cell areas. Boxes show 25th, 50th, and 75th centiles; whiskers indicate $25^{th}/75^{th}$ centiles +/- 1.5 × interquartile range. For **b-d**, beneath boxes are absolute numbers of patients in each category.

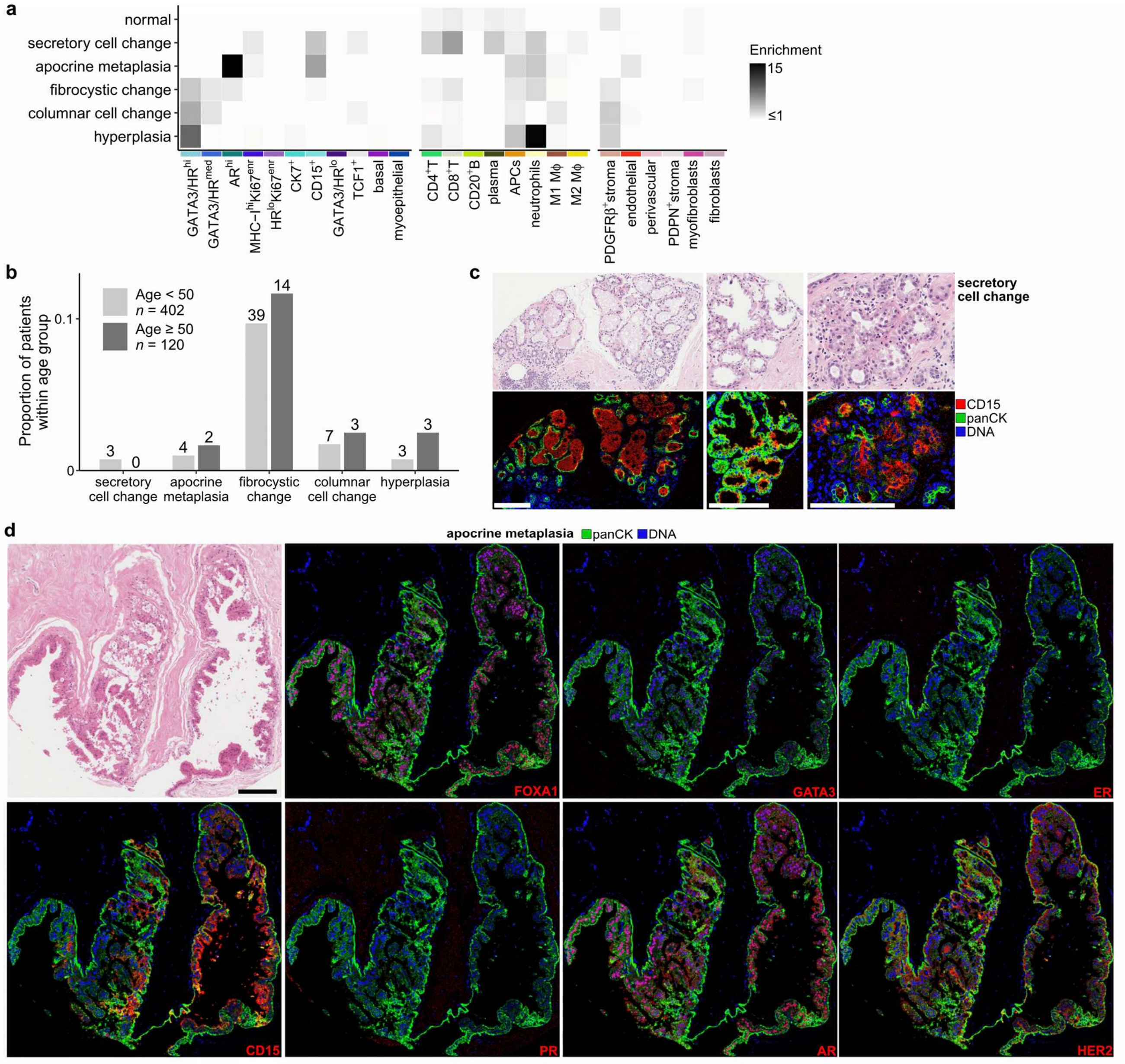

Supplementary Fig. 8|Breast histological and molecular changes. a, Enrichment heatmap of phenotypes (*x* axis) in histological categories (*y* axis). Enrichments are equivalent to the ratio of observed counts to expected counts; expected counts are based on the proportion of cells in each phenotype and histological category. Only enrichments ≥ 1 are plotted. **b**, Bar plot of the presence of histological changes in patients younger and older than 50 years. **c**, Representative secretory cell change images. **d**, Representative apocrine metaplasia image. All scale bars, 150 μm.

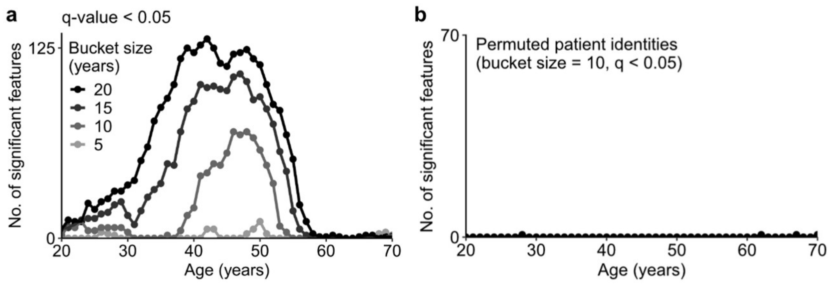

Supplementary Fig. 9|Window size selection and permutation of patient identities. a, Number of significant features as a function of age, with different window (bucket) sizes and a q-value significance cutoff of 0.05. **b**, As in **a**, with randomly-permuted patient identities at a bucket size of 10 years and q-value cutoff of 0.05, showing loss of significance.
