# Supplementary material for: Single-cell spatial atlas of the aging human breast
Source: Nat Aging. 2026 Mar 31;6(4):916–31. doi: 10.1038/s43587-026-01104-3 (PMC13099655; doi:10.1038/s43587-026-01104-3)
Supplement: Supplementary file 2 — Reporting Summary [file 43587_2026_1104_MOESM2_ESM.pdf]

nature portfolio

Corresponding author(s): Samuel Aparicio, H Raza Ali

Last updated by author(s): Feb 23, 2026

Reporting Summary

Nature Portfolio wishes to improve the reproducibility of the work that we publish. This form provides structure for consistency and transparency in reporting. For further information on Nature Portfolio policies, see our Editorial Policies and the Editorial Policy Checklist.

Statistics

For all statistical analyses, confirm that the following items are present in the figure legend, table legend, main text, or Methods section.

n/a	Confirmed	
☐	☒	The exact sample size (*n*) for each experimental group/condition, given as a discrete number and unit of measurement
☐	☒	A statement on whether measurements were taken from distinct samples or whether the same sample was measured repeatedly
☐	☒	The statistical test(s) used AND whether they are one- or two-sided<br>*Only common tests should be described solely by name; describe more complex techniques in the Methods section.*
☐	☒	A description of all covariates tested
☐	☒	A description of any assumptions or corrections, such as tests of normality and adjustment for multiple comparisons
☐	☒	A full description of the statistical parameters including central tendency (e.g. means) or other basic estimates (e.g. regression coefficient) AND variation (e.g. standard deviation) or associated estimates of uncertainty (e.g. confidence intervals)
☐	☒	For null hypothesis testing, the test statistic (e.g. *F*, *t*, *r*) with confidence intervals, effect sizes, degrees of freedom and *P* value noted<br>*Give P values as exact values whenever suitable.*
☒	☐	For Bayesian analysis, information on the choice of priors and Markov chain Monte Carlo settings
☒	☐	For hierarchical and complex designs, identification of the appropriate level for tests and full reporting of outcomes
☐	☒	Estimates of effect sizes (e.g. Cohen's *d*, Pearson's *r*), indicating how they were calculated

Our web collection on statistics for biologists contains articles on many of the points above.

Software and code

Policy information about availability of computer code

Data collection	Aperio eSlideManager (Leica Biosystems) - viewing H&E slides and making annotations<br>CyTOF Software v7.0 (Fluidigm) - for collection of data from the Hyperion+ imaging mass cytometer

Data analysis	Image processing:<br>1. imctools package (https://github.com/BodenmillerGroup/imctools)- for conversion of .txt files to .tiff files<br>2. DeepCell package (https://github.com/vanvalenlab/deepcell-tf) - for single-cell segmentation<br>3. CellProfiler v4.0.6 (https://cellprofiler.org) - generating single cell measurements and vessel/myoepithelial layer masks<br>4. Ilastik v1.3 - training vessel/myoepithelial layer masks<br><br>Custering:<br>1. Rphenograph/Rphenoannoy (https://github.com/JinmiaoChenLab/Rphenograph, https://github.com/stuchly/Rphenoannoy) - for single-cell clustering<br>2. CellCharter (https://github.com/CSOgroup/cellcharter) - for spatial clustering<br><br>Statistical analysis:<br>R v4.2.3; python v3.11.4<br>1. R spatstat (https://spatstat.org/) - for spatial analyses<br>2. igraph (https://r.igraph.org/) - for network analysis<br><br>All code used to reproduce the figures is available at https://github.com/HRazaAliLab/IMCBreastAgeingAtlas.

For manuscripts utilizing custom algorithms or software that are central to the research but not yet described in published literature, software must be made available to editors and reviewers. We strongly encourage code deposition in a community repository (e.g. GitHub). See the Nature Portfolio guidelines for submitting code & software for further information.

Data

Policy information about availability of data

All manuscripts must include a data availability statement. This statement should provide the following information, where applicable:

- Accession codes, unique identifiers, or web links for publicly available datasets
- A description of any restrictions on data availability
- For clinical datasets or third party data, please ensure that the statement adheres to our policy

All data, including counts of cell phenotypes and interaction data are available in a Zenodo data repository (https://doi.org/10.5281/zenodo.18418221). Other data generated during the study are available from the authors upon reasonable request.

Research involving human participants, their data, or biological material

Policy information about studies with human participants or human data. See also policy information about sex, gender (identity/presentation), and sexual orientation and race, ethnicity and racism.

Reporting on sex and gender	The normal breast TMA only contains female cases.
Reporting on race, ethnicity, or other socially relevant groupings	No metadata is included other than age.
Population characteristics	The normal breast TMA used all available cases collected in a large biobank series at BC Cancer from outside sources conducting breast reduction mammoplasty, so it represents a sample of convenience. Age was the only relevant parameter included.
Recruitment	Patients were recruited by research assistants explaining the value of donated normal breast tissue material for research purposes in discovering additional treatment options, and consent obtained with oversight of research ethics boards.
Ethics oversight	Patient specimens were obtained from the BC Cancer/University of British Columbia Research Ethics Board protocol titled “BC Cancer Mammary Cell Bank” under ethics approval H19-03798 which provides for informed consent or waiver of consent for secondary research use of anonymized legacy biobank human tissues surplus to diagnostic requirement. Tissue analysis with these specimens was conducted under protocols H25-01125 and H19-03794.

Note that full information on the approval of the study protocol must also be provided in the manuscript.

Field-specific reporting

Please select the one below that is the best fit for your research. If you are not sure, read the appropriate sections before making your selection.

☒ Life sciences ☐ Behavioural & social sciences ☐ Ecological, evolutionary & environmental sciences

For a reference copy of the document with all sections, see nature.com/documents/nr-reporting-summary-flat.pdf

Life sciences study design

All studies must disclose on these points even when the disclosure is negative.

Sample size	Normal breast TMA: 537 cancer-free breast reduction mammoplasty cases. The data set size is sufficiently large to clearly detect age-correlated patterns of a biologically relevant size with sufficiently small p-values including with adjustment for multiple comparisons.
Data exclusions	Some tissues were not assessed by IMC due to either having no tissue available for sampling, or no areas of epithelium available to image. As each acquired image was manually checked, we excluded images that had poor data quality (very few cells, poor staining, bad tissue quality) as these were not representative of the tissue microenvironment. No other data points/outliers have been excluded in our subsequent analyses.
Replication	A second study was not conducted to replicate findings.
Randomization	Normal breast TMA case samples were randomly distributed across TMA slides during construction.
Blinding	Blinding in the traditional statistical sense was not relevant for this study, as this study was an assessment of spatial characteristics exhibiting age-associated effects rather than a comparison of groups.

Reporting for specific materials, systems and methods

We require information from authors about some types of materials, experimental systems and methods used in many studies. Here, indicate whether each material, system or method listed is relevant to your study. If you are not sure if a list item applies to your research, read the appropriate section before selecting a response.

Materials & experimental systems

n/a	Involved in the study
☐	☒ Antibodies
☒	☐ Eukaryotic cell lines
☒	☐ Palaeontology and archaeology
☒	☐ Animals and other organisms
☐	☒ Clinical data
☒	☐ Dual use research of concern
☒	☐ Plants

Methods

n/a	Involved in the study
☒	☐ ChIP-seq
☒	☐ Flow cytometry
☒	☐ MRI-based neuroimaging

Antibodies

Antibodies used	A full list of all antibodies used along with their staining concentrations can be found in Supplementary Table 1.
Validation	All antibodies were validated firstly in autofluorescence and then again after conjugation to a metal antibody. The tissues used were commercial human tissue that would be expected to express the target protein. Further details are available in the Methods section.

Clinical data

Policy information about clinical studies

All manuscripts should comply with the ICMJE guidelines for publication of clinical research and a completed CONSORT checklist must be included with all submissions.

Clinical trial registration	This is an observational study conducted using data previously collected from tissue material in medical research studies, rather than from clinical trials.
Study protocol	Since none of the data were obtained from any clinical trial, there are no study protocols.
Data collection	Tissue is from a large biobank series at BC Cancer from outside sources conducting breast reduction mammoplasties.
Outcomes	Observational metadata are described in the supplemental material associated with the publication for the cited study from which our data were drawn.

Plants

Seed stocks	N/A
Novel plant genotypes	N/A
Authentication	N/A
